# Genomic Analysis of G2P[4] Group A Rotaviruses in Zambia Reveals Positive Selection in Amino Acid Site 7 of Viral Protein 3

**DOI:** 10.3390/v15020501

**Published:** 2023-02-11

**Authors:** Peter N. Mwangi, Robyn-Lee Potgieter, Julia Simwaka, Evans M. Mpabalwani, Jason M. Mwenda, Milton T. Mogotsi, Nonkululeko Magagula, Mathew D. Esona, A. Duncan Steele, Mapaseka L. Seheri, Martin M. Nyaga

**Affiliations:** 1 Next Generation Sequencing Unit and Division of Virology, Faculty of Health Sciences, University of the Free State, Bloemfontein 9300, South Africa; 2 Institute of Basic and Biomedical Sciences, Department of Biomedical Sciences, The Levy Mwanawasa Medical University, Lusaka 10101, Zambia; 3 Department of Paediatrics and Child Health, School of Medicine, University of Zambia, Ridgeway, Lusaka RW50000, Zambia; 4 World Health Organization, Regional Office for Africa, Brazzaville P.O. Box 06, Congo; 5 Diarrheal Pathogens Research Unit, Faculty of Health Sciences, Sefako Makgatho Health Sciences University, Pretoria 0204, South Africa

**Keywords:** G2P[4], rotavirus, Zambia, whole-genome sequencing, DS-1-like genotype constellation

## Abstract

The G2P[4] genotype is among the rotavirus strains that circulate commonly in humans. Several countries have reported its immediate upsurge after the introduction of rotavirus vaccination, raising concern about sub-optimal vaccine effectiveness against this genotype in the long term. This study aimed to gain insight into the evolution of post-vaccine Zambian G2P[4] group A rotavirus (RVA) strains and their overall genetic make-up by analysis of sequence alignments at the amino acid (AA) level. Twenty-nine Zambian G2P[4] rotavirus strains were subjected to whole-genome sequencing using the Illumina MiSeq^®^ platform. All the strains exhibited the typical DS-1-like genotype constellation, and the nucleotide sequences of the 11 genome segments showed high nucleotide similarities (>97%). Phylogenetic analyses together with representative global G2P[4] RVA showed that Zambian strains clustered into human lineages IV (for VP2, VP4, VP7, NSP1, and NSP5), V (for VP1, VP3, VP6, NSP2, and NSP3), and XXIII (for NSP4). The AA differences between the lineages where the study strains clustered and lineages of global reference strains were identified and analyzed. Selection pressure analysis revealed that AA site seven in the Viral Protein 3 (VP3) genome segment was under positive selection. This site occurs in the region of intrinsic disorder in the VP3 protein, and Zambian G2P[4] strains could potentially be utilizing this intrinsically disordered region to survive immune pressure. The Zambian G2P[4] strains from 2012 to 2016 comprised the G2P[4] strains that have been circulating globally since the early 2000s, highlighting the epidemiological fitness of these contemporary G2P[4] strains. Continuous whole-genome surveillance of G2P[4] strains remains imperative to understand their evolution during the post-vaccination period.

## 1. Introduction

Group A rotavirus (RVA)-induced acute gastroenteritis persists among the leading causes of mortality in children under five years, particularly in sub-Saharan Africa and southeast Asia [1]. To alleviate RVA disease burden, the World Health Organization (WHO) prequalified four rotavirus vaccines, namely Rotarix^®^ (GlaxoSmithKline, Rixenstart, Belgium), RotaTeq^®^ (Merck & Co., Whitehouse Station, NJ, USA), Rotavac^®^ (Bharat Biotech, India), and Rotasiil^®^ (Serum Institute, India), after extensive efficacy and safety studies (https://www.who.int/publications/i/item/WHO-IVB-2021.03 (accessed on 10 January 2023)). Vaccination and, to an extent, improvement in sanitation have substantially reduced RVA disease mortality cases from ~500,000 during the pre-vaccination era to ~128,000 during the post-vaccination period [1,2]. In Zambia, the Rotarix^®^ vaccine was introduced in January 2012 in Lusaka as a pilot project and later rolled out countrywide in November 2013 [3,4], with vaccine coverage of 87% in 2021 (https://immunizationdata.who.int/pages/profiles/zmb.html (accessed on 17 January 2023)). Rotavirus vaccination has been shown to have a significant impact on rotavirus hospitalizations and deaths in Africa [5] although the widespread use of RVA vaccines has raised concerns about vaccine-induced selective pressure on circulating RVA strains, which may result in putative vaccine-escape mutants that may affect the effectiveness of RVA vaccines in the long term [6].

Rotavirus belongs to the *Sedoreoviridae* family of the order *Reovirales* and comprises 11 genome segments [7]. The ~18 kb rotavirus genome is organized into six structural proteins, namely viral proteins (VP1–4, 6, and 7) and five non-structural proteins (NSP1–5) [7]. The capsid proteins, VP7 (denoted G for its glycoprotein nature) and VP4 (denoted P due to its protease sensitivity), act as neutralizing antigens and are traditionally used to classify rotavirus strains into a G/P binomial scheme [8]. The G1P[8], G2P[4], G3P[8], G4P[8], G9P[8], and G12P[8] are the most commonly identified RVA genotype combinations in humans [9]. To fully describe rotavirus strains, a whole-genome-based classification scheme based on the genotype of each of the 11 genome segments is preferred and has revealed two major genotype constellations: a Wa-like constellation called genogroup one and a DS-1-like constellation called genogroup two [10]. The whole-genome classification nomenclature of RVA is Gx-P[x]-Ix-Rx-Cx-Mx-Ax-Nx-Tx-Ex-Hx, representing the VP7 (**G**lycoprotein, G), VP4 (**P**rotease sensitive, P), VP6 (**I**ntermediate, I), VP1 (RNA dependent **R**NA polymerase, R), VP2 (**C**ore, C), VP3 (**M**ethyltransferase, M), NSP1 (**A**ntagonist, A), NSP2 (**N**Tpase, N), NSP3 (**T**ranslation enhancer, T), NSP4 (**E**nterotoxin, E), and NSP5 (p**H**osphoprotein, H) genome segments, respectively, with the letters describing the properties of the 11 genome segments and x indicating the numbers of the corresponding genotypes [10].

The G1P[8], G3P[8], G9P[8], and G12P[8] typically belong to the Wa-like genogroup [9]. However, atypical DS-1-like G1P[8] [11,12], G3P[8] [13,14], and G9P[8] strains [15] have been reported. On the other hand, the G2P[4] strains usually belong to the DS-1-like genogroup [16]. The rotavirus genome has a segmented nature that allows for reassortment events, leading to the emergence of reassortant RVA strains [17,18,19,20]. Additionally, the rotavirus RNA-dependent RNA polymerase, due to its error-prone nature, causes the buildup of sequential point mutations [7]. The varying mutation rates among different RVA genome segments reflect the different selective pressures exerted on different RVA genome segments [12,21,22]. The vast diversity of rotavirus strains is demonstrated by the 42 G, 58 P, 32 I, 28 R, 24 C, 24 M, 39 A, 28 N, 28 T, 32 E, and 28 H genotypes reported at the time of writing this work (https://rega.kuleuven.be/cev/viralmetagenomics/virus-classification/rcwg (accessed on 17 January 2023)).

The G2P[4] genotype has been observed to increase or maintain dominance after the introduction of the monovalent G1P[8] rotavirus vaccine in several countries such as Australia, Belgium, Brazil, Botswana, Japan, Kenya, Malawi, Saudi Arabia, South Africa, and Venezuela [6,23,24,25,26,27,28,29,30,31]. Although rotavirus vaccines offer cross-protection against different rotavirus genotypes, there is potential for differential vaccine effectiveness against strains from different genogroups such as G2P[4] [32]. As a result, it is imperative to elucidate the diversity and genetic evolution of G2P[4] rotaviruses, especially at the whole-genome level. Whole-genome studies of G2P[4] strains conducted in Italy and Japan reported a genetic shift in the global G2P[4] strains in the early 2000s [33,34]. A long-scale study looking into G2P[4] strains collected seven years before and seven years after rotavirus vaccine introduction in South Africa reported pre- and post-vaccination sub-lineages defined by AA substitutions observed outside known antigenic regions [35].

In Zambia, a four-year G2P[4] prevalence period post Rotarix^®^ introduction was reported by surveillance based on conventional VP7 and VP4 genotyping [36]. Apart from the whole-genome sequencing report of a human-porcine reassortant G5P[6] strain and four intergenogroup reassortant strains [17,19], there is generally a paucity of published Zambian whole-genome G2P[4] RVA sequence data. The purpose of the present study was to perform whole-genome characterization of Zambian G2P[4] strains collected during routine surveillance to gain insight into their overall genetic makeup and evolution.

## 2. Materials and Methods

### 2.1. Ethics Statement

The Health Sciences Research Ethics Committee (HSREC) at the University of the Free State in Bloemfontein, South Africa, granted ethical permission for this study under ethics number (UFS-HSD2016/1082).

### 2.2. Stool Specimen and Strain Description

RVA-positive fecal specimens (*n* = 133) were obtained from children under the age of five years presenting with acute gastroenteritis as part of the ongoing World Health Organization Regional Office for Africa (WHO-AFRO) RVA surveillance program in Zambia. The stool samples were collected between 2011–2016 and were characterized into G and P types by conventional genotyping methods at the Diarrheal Pathogens Research Unit (DPRU), a WHO Rotavirus Reference Laboratory in South Africa (WHO RRL SA). Briefly, viral dsRNA was extracted from 10% fecal suspension using the QIAmp viral RNA extraction method (Qiagen, Hilden, Germany). The extracted RNA was reverse transcribed and amplified using consensus primer pairs Con2/Con3 and sBeg/End9 as described previously [37,38]. The resulting cDNA template was used for G and P typing using semi-nested RT-PCR amplification of the genes targeting VP7 and VP4. Out of the 133 samples, 29 were G2P[4] strains that were analyzed in this study.

### 2.3. Data Collection from GenBank

Datasets from different global geographical regions retrieved from GenBank were included for analysis (The accession numbers for the reference sequences used in this study are included as Supplementary Data S1).

### 2.4. Double-Stranded RNA Extraction

Rotavirus dsRNA was extracted as previously described [20]. Briefly, ~100 mg of fecal specimen was added to 200 µL of phosphate-buffered saline (PBS) solution, 0.01 M, pH (Sigma-Aldrich^®^, St Louis, MO, USA) to generate a fecal suspension. The fecal suspension was vortexed for 10 s and then left to stand for ten minutes at room temperature, after which a 300 µL of fecal suspension was added to 900 µL of TRI-Reagent^®^-LS (Molecular Research Center, Cincinnati, OH, USA). The fecal-TRI-Reagent solution was vortexed for 10 s and left to stand at room temperature for 10 min. A 300 µL volume of chloroform (Sigma-Aldrich^®^, St Louis, MO, USA) was added to the fecal-Tri-Reagent solution. After vortexing the solution for 10 s and letting it stand for 5 min at room temperature, the solution was centrifuged for 18,000× *g* for 20 min at 4 °C. The aqueous supernatant was removed and precipitated with 700 µL of ice-cold isopropanol (Sigma-Aldrich^®^, St Louis, MO, USA). Extracted RNA was incubated in 8 M Lithium chloride (Sigma-Aldrich^®^, St Louis, MO, USA) for 16 h at 4 °C to enrich for rotavirus dsRNA and then subsequently purified using the MinElute PCR purification kit (Qiagen, Hilden, Germany). Electrophoresis was then performed on a 5 µL aliquot of dsRNA in 1% 0.5 TBE agarose (Bioline, UK) gel stained with Pronasafe (Condalab, UK) for 1 h at 95 volts to check the integrity of the extracted and purified rotavirus dsRNA.

### 2.5. cDNA Synthesis

The complementary DNA (cDNA) was synthesized using the Maxima H Minus Double-Stranded cDNA Synthesis Kit (Thermo Fischer Scientific, Waltham, MA, USA), with modifications. Briefly, 13 µL of extracted viral RNA was denatured at 95 °C for 5 min, and then, 1 µL of random hexamer primer was added. Annealing was performed at 65 °C for 5 min. A 5 µL volume of First-Strand Reaction Mix was added to the solution, followed by 1 µL of First-Strand Enzyme. The mixture was incubated for 10 min at 25 °C, followed by 2 h at 50 °C. Afterward, the following components were added: 55 µL of nuclease-free water, 20 µL of Second-Strand Reaction Mix, and 5 µL of Second-Strand Enzyme, and the solution was incubated at 16 °C for 60 min. A 6 µL volume of 0.5 M EDTA, pH 8.0 was added to stop the reaction, and residual RNA was removed by adding 10 µL of RNase I.

### 2.6. DNA Library Preparations and Whole-Genome Sequencing

DNA libraries were generated using the Nextera XT DNA Library Preparation Kit (Illumina, San Diego, CA, USA) as per the manufacturer’s instructions. Briefly, the genomic cDNA was tagmented, indexed, and then amplified. Afterward, the amplified, tagmented, and indexed DNA was cleaned up using Ampure XP beads (Beckman Coulter, Pasadena, CA, USA). The library quality and fragment sizes were then validated using an Agilent 2100 Bioanalyzer (Agilent Technologies, Waldbronn, Germany). The DNA libraries were then normalized to 4 nM, and then, 5 µL of normalized libraries were then pooled together into a single tube. A 5 µL pool of the normalized libraries was denatured using 5 µL of 0.2 N sodium hydroxide. Afterward, 990 µL of hybridization buffer was added to the 10 µL of the denatured 4 nM DNA library to dilute to 20 pM with a further dilution to achieve a final loading concentration of 8 pM with a 20% Phix control. The combined library and Phix were loaded into the Illumina V3 reagent cartridge and sequencing performed for 600 cycles (301 bp X 2 paired-end) on a MiSeq^®^ Illumina platform (Illumina, San Diego, CA, USA).

### 2.7. Genome Assembly

Quality control analysis of the sequenced raw data was performed using FASTQC v.0.11.9 [39]. Adapter sequences were trimmed from the raw FASTQ sequence data using BBDuk trimmer (https://sourceforge.net/projects/bbmap/ (accessed on 27 December 2022)). Reference-based mapping using the prototype DS-1-like reference strain (accession numbers HQ650116-HQ650126) was performed using Geneious Read Mapper 6.0.3 [40]. The Geneious Consensus Tool was used for consensus calling [40]. The Annotate and Predict Tool in Geneious Prime^®^ version 2020.1.1 was used to annotate low coverage (<200) regions, whereby a coverage-annotated track indicating the annotated regions was generated to aid in consensus calling.

### 2.8. Whole-Genome Genotyping

The genome segments were genotyped using the web-based Virus Pathogen Database and Analysis Resource (ViPR) to generate full-genome constellations [41].

### 2.9. Phylogenetic Analysis

Basic Local Alignment Search Tool (BLAST) and the National Center for Biotechnology Information’s (NCBI) Virus Variation Resource were used to compile reference sequences [42,43]. The phylogenetic trees for the genome segments encoding VP4 and VP7 were created using previously described lineage designations [35,44]. A previously proposed lineage framework was used to assign lineages to non-G and non-P genome segments [45].

The open reading frame (ORF) sequences for each genome segment were aligned using the Multiple Sequence Comparison by Log-Expectation (MUSCLE) tool [46] in Molecular Evolutionary Genetic Analysis (MEGA) version X [47]. The Model Test in MEGA X was used to estimate the best evolutionary model. The evolutionary models used were Generalized Time Reversible + Gamma + Inversions (GTR + G + I) for VP1, VP2, and VP3; Tamura-3-parameter + Gamma + Inversions (T92 + G + I) for NSP2, NSP3, and NSP4; Tamura-3-parameter + Gamma (T92 + G) for VP4 and VP6; and Tamura-3 parameter + Inversions (T92 + I) for VP7, NSP1, and NSP5. For model selection analysis, maximum likelihood was chosen as the statistical method, and gaps/missing data were handled using partial deletion with an 80% site coverage cut-off and moderate-branch swap filtering. The MEGA X software was used to generate maximum likelihood phylogenetic trees for each genome segment with a 1000 bootstrap support.

### 2.10. Inference of Selective Pressures

In order to enhance the estimation of sites in the genome of G2P[4] RVA strains undergoing negative and positive selection, we employed three evolutionary analyses method available from the DataMonkey webserver [48]. These are fixed-effects likelihood (FEL) [49], Fast Unbiased Bayesian AppRoximation (FUBAR) [50], and Mixed Effects Model of Evolution (MEME) [51]. The basis of these three selective pressure analyses methods are the estimation of non-synonymous (dN) and synonymous (dS) substitution rates on a per-site basis for the coding alignment of the G2P[4] RVA genome segments. The FEL and MEME methods utilize a maximum likelihood approach, while FUBAR employs a Bayesian approach [48]. The FEL and MEME analysis was performed with *p*-value threshold of 0.1, while FUBAR was performed with a posterior probability of 0.9.

## 3. Results

### 3.1. Whole Genotype Analysis

All the 29 study strains had a pure DS-1-like genotype constellation (G2-P[4]-I2-R2-C2-M2-A2-N2-T2-E2-H2). The genome segments from the 29 G2P[4] strains showed nucleotide sequence identities of >97% amongst each other (Supplementary Data S2).

### 3.2. Phylogenetic and Sequence Analysis

Phylogenetic analyses together with representative global G2P[4] RVAs showed that Zambian strains clustered into human lineages IV (for VP4, VP7, VP2, NSP1, and NSP5), V (for VP1, VP3, VP6, NSP2, and NSP3), and XXIII (for NSP4) (Figure 1, Figure 2, Figure 3, Figure 4, Figure 5, Figure 6, Figure 7, Figure 8, Figure 9, Figure 10 and Figure 11).

**Figure 1:**
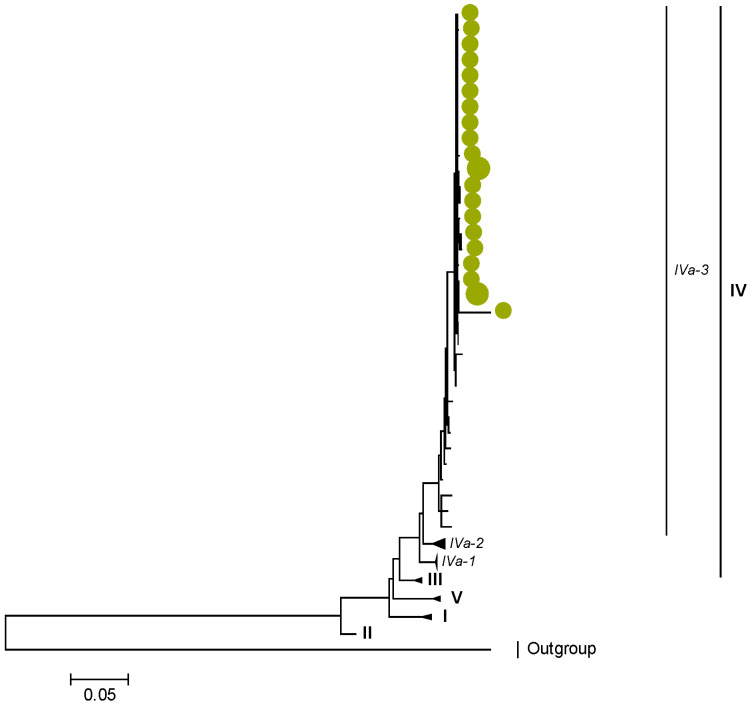
VP7. Maximum Likelihood phylogenetic tree of the genome segment encoding VP7 of Zambian G2P[4] sequences. The study strains are indicated with light-green circular symbols. Lineages are indicated in Roman numerals, while sub-lineages are in italic. The scale number indicates the number of nucleotide substitutions per site.

Zambian G2 sequences clustered further into sub-lineage IVa_3 (Figure 1). The AA substitutions (I44M, D96N, S178N, and I287V) were observed between G2 strains in lineages I, II, III, and V in respect to the G2 strains in lineage IV (Table 1 and Table 2).

**Figure 2:**
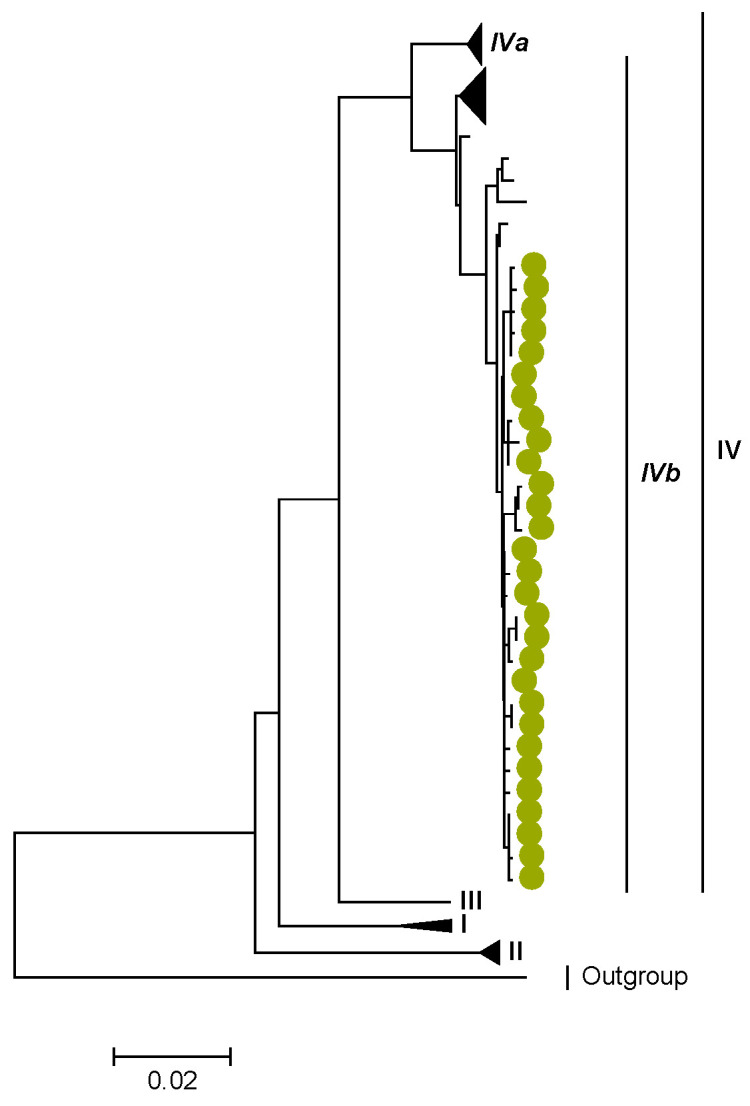
VP4. Maximum Likelihood phylogenetic tree of the genome segment encoding VP4 of Zambian G2P[4] sequences. The study strains are indicated with light-green circular symbols. Lineages are indicated in Roman numerals, while sub-lineages are in italic. The scale number indicates the number of nucleotide substitutions per site.

We identified AA substitutions (I20V, S598L, and M630I) between P[4] strains in lineages I, II, and III in respect to the P[4] strains in lineage IV, where the study P[4] sequences clustered (Table 3 and Table 4). Within lineage IV, Zambian P[4] sequences further clustered into sub-lineage IVb (Figure 2).

Amino acid position 159 of lineage V of R2 sequences where Zambian strains clustered featured an arginine, while other R2 lineages had a lysine (Tables S1 and S2). Three AA differences (N/D28I, I70V, and V538I) were observed between C2 sequences in lineages I–III and V–XIV in respect to lineage IV, where the study strains clustered (Tables S3 and S4). We observed three AA differences (S49N, V209I, and M348V) for the VP3 sequences between lineages I–V and VI–XIV in respect to the lineage V, where Zambian sequences grouped (Tables S5 and S6).

In the genome segment encoding NSP1, three AA differences were observed between the lineages I–II, V, and the lineage IV of the study strains. These differences are represented by l273F, H319R, and K429R, as shown in Tables S7 and S8. Similarly, in NSP2, NSP3, NSP4, and NSP5, there were conservative AA differences between the reference lineages and the lineage of the study strains. These differences are represented by L273F in NSP2, M61I in NSP3, R137Q in NSP4, and V122M in NSP5, as shown in Tables S9–S16.

### 3.3. Selection Pressure Analysis

**Figure 12:**
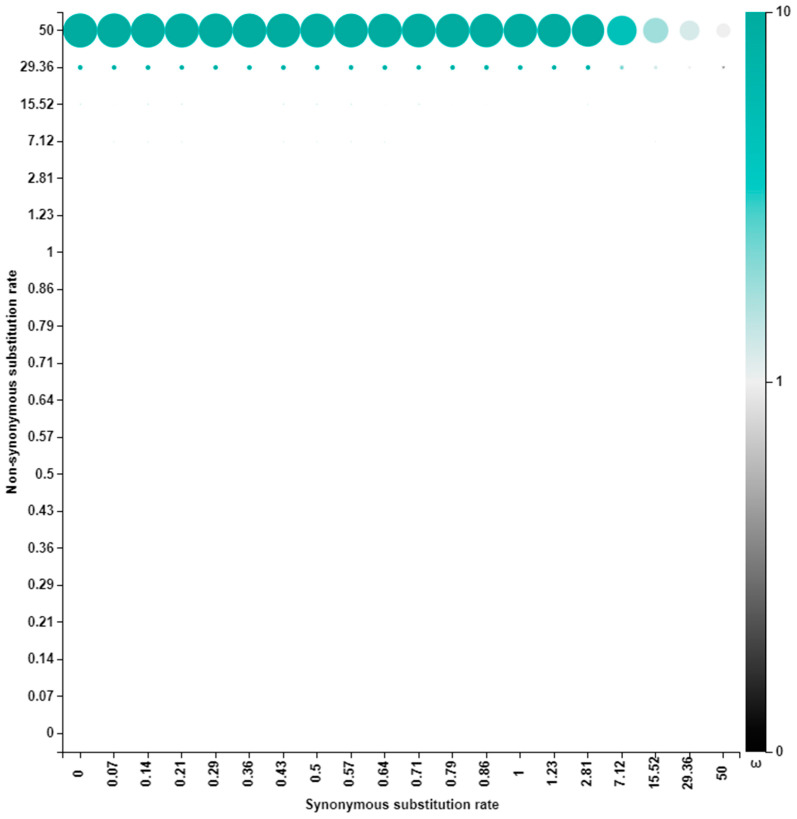
Posterior distributions of VP3 site seven over the discretized rate grid. The size of a dot is proportional to the posterior weight allocated to that grid point, and the color shows the intensity of selection.

The AA sites in the genome segments of Zambian G2P[4] strains were undergoing purifying selection with the exception of the AA site seven in the VP3 gene, which was under diversifying/positive selection (Figure 12 and Table S17).

This site was found to have two AA: isoleucine occurring in ~40% of the strains and arginine in ~60% of the strains (Table S18).

## 4. Discussion

The study reports on whole-genome analysis of 29 G2P[4] RVA strains collected from Zambia from 2011 to 2016. All the Zambian G2P[4] gene sequences exhibited a pure DS-1-like constellation, high nucleotide similarities (>97%), and clustered alongside contemporary human G2P[4] lineages. In essence, Zambian G2P[4] strains emerged independent of interspecies-transmission events, suggesting they were of human origin. These findings are consistent with whole-genome studies of G2P[4] studies in several countries, whereby the reported G2P[4] strains evolved devoid of reassortment with animal genes [23,44,52,53].

The Zambian G2 sequences, which were all post-vaccine, clustered into sub-lineage IVa_3, which is relatable to a South African study wherein post-vaccine G2 strains clustered in sub-lineage IVa_3, while pre-vaccine strains clustered in sub-lineage IVa_1 [35]. In that study, G2 sequences in sub-lineage IVa_3 were found to have a serine at position 15, while sequences in sub-lineage IVa_1 featured phenyalanine, indicating that the AA substitution could be of epidemiological relevance. The aspartate to asparagine substitution at escape mutation site 96, which hallmarks the G2 sequences from the year 2000 [44], could be a selective advantage to enable antibody-dependent enhancement, potentially favoring the production of non-neutralizing antibodies to this protein region [54]. Therefore, this AA site possibly plays a significant role in epidemiological fitness and consequent transmissibility of lineage IV G2 strains. Methionine identification at the Cytotoxic T lymphocyte (CTL) region [55] of lineage IV G2 sequences could have far-reaching biological implications. Apart from protein initiation, methionine’s unbranched side chain provides energetic stability, leading to different conformations [56]. Additionally, the presence of the sulfur atom makes methionine an optimal AA for transient protein–protein interactions [57] that could interfere with the clearance of rotavirus infection.

According to the literature, the isoleucine substitution for valine residue is suggested to shift the equilibrium to a more compact conformation [58]. Therefore, the occurrence of this AA substitution at the hemagglutination site 120 [59] of Zambian P[4] lineage IV strains could be posing possible functional changes such as improving interaction with the sialic-acid-containing structure during cell attachment. Notably, the P[4] study sequences were found to cluster in sub-lineage IVb. This is similar to what was observed in a South African study of post-vaccine P[4] strains [35]. The South African study reported that post-vaccine P[4] sequences in sub-lineage IVb had an arginine at position 162, while pre-vaccine P[4] sequences had a glycine. The epidemiological relevance of this radical AA substitution warrants more exploration as to whether it contributes to epidemiological fitness of the contemporary circulating G2P[4] strains. The radical AA substitution of serine for leucine from polar to nonpolar residue [60] at position 598 of the VP5 portion could be enhancing the permeabilization effect of membranes to enable VP4 entry into the host cell [61], as the presence of the bulkier hydrophobic leucine side chain induces local rearrangements that may likely induce small but significant differences in function [60]. Although we noted that most of the AA differences between the lineages in the rest of the genome segments were conservative, we posit that the intrinsic link between the AA ambiguities and rotavirus protein multifunctionality requires in-depth exploration to comprehensively understand the epidemiological fitness of the lineages in the post-vaccination context era.

Most AA sites were undergoing purifying selection, probably as a strategy to purge deleterious polymorphisms that arise due to the error-prone nature of the RNA polymerase enzyme [7]. However, AA site seven in the VP3 was under positive selection. Arginine and isoleucine that were observed in this site have extreme physicochemical disparities, whereby arginine is a polar positively charged residue, while isoleucine is a non-polar neutrally charged residue [62]. The AA residues under strong selective pressure are suggested to have essential catalytic functions [63]. Therefore, this AA site is likely selected to enhance the methylase capping activity of the VP3. Additionally, apart from lying in the signal sequence domain region, site seven was also found among the disordered protein regions of VP3 [64]. Viruses utilize intrinsically disordered regions to survive in harsh environments to evade the host immune system and to hijack and manipulate host cellular proteins [65]. Therefore, selection for this effect could be at play in this region.

Our analysis would have been more enriched with analysis performed in the context of pre-vaccination Zambian G2P[4] samples. However, natural fluctuations are known to impact the prevalence of circulating rotavirus strains [53], and sampling for the current study was performed based on availability. Regardless, our study findings provide valuable whole-genome insights regarding the evolution of G2P[4] strains in Zambia.

## 5. Conclusions

The G2P[4] strains circulating in Zambia from 2012 to 2016 belong to the lineages of G2P[4] strains that have been circulating globally since the early 2000s, highlighting the epidemiological fitness of these contemporary strains. The analysis of AA substitutions defining the lineages of the circulating G2P[4] strains provides an insightful perspective to understanding their evolution.

## Figures and Tables

**Figure 3:**
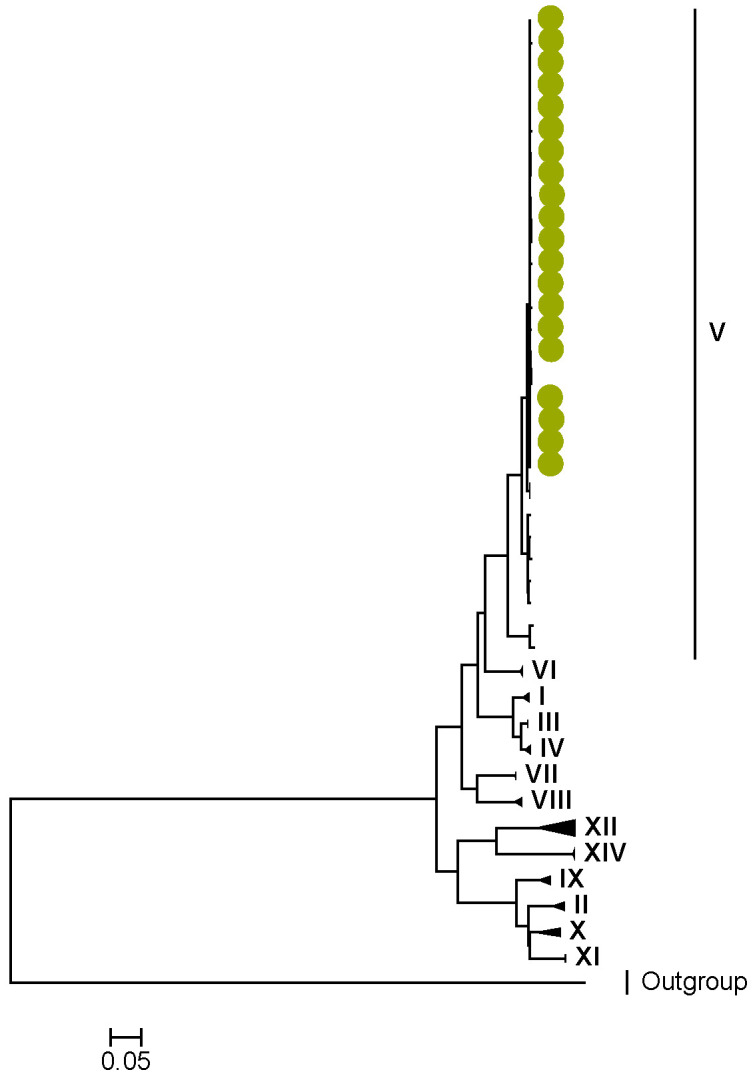
VP1. Maximum Likelihood phylogenetic tree of the genome segment encoding VP1 of Zambian G2P[4] sequences. The study strains are indicated with light-green circular symbols. Lineages are indicated in Roman numerals. The scale number indicates the number of nucleotide substitutions per site.

**Figure 4:**
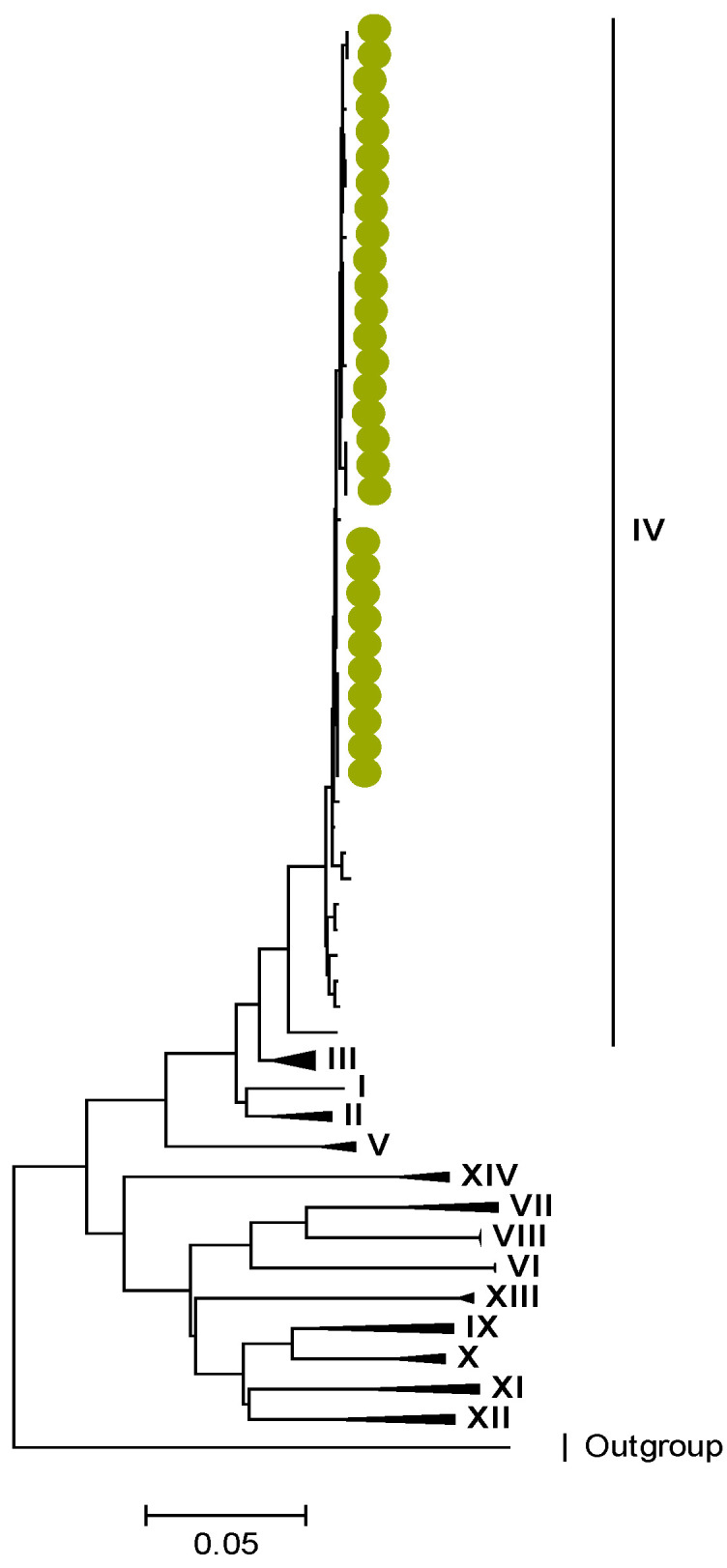
VP2. Maximum Likelihood phylogenetic tree of the genome segment encoding VP2 of Zambian G2P[4] sequences. The study strains are indicated with light-green circular symbols. Lineages are indicated in Roman numerals. The scale number indicates the number of nucleotide substitutions per site.

**Figure 5:**
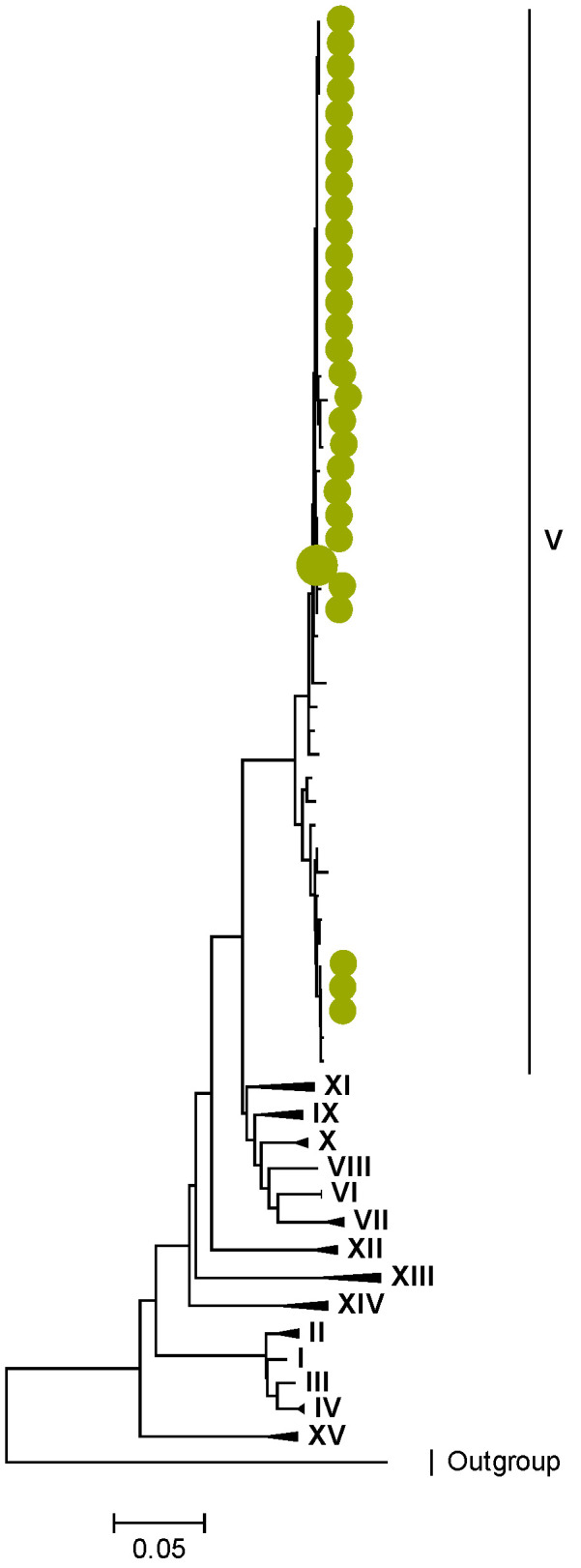
VP3. Maximum Likelihood phylogenetic tree of the genome segment encoding VP3 of Zambian G2P[4] sequences. The study strains are indicated with light-green circular symbols. Lineages are indicated in Roman numerals. The scale number indicates the number of nucleotide substitutions per site.

**Figure 6:**
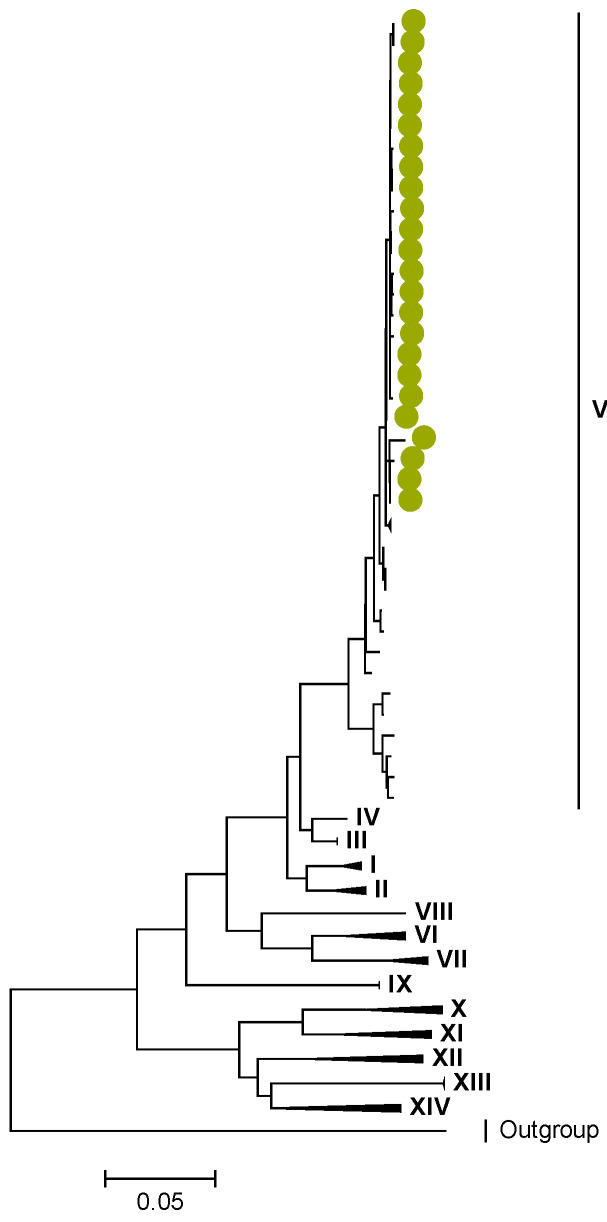
VP6. Maximum Likelihood phylogenetic tree of the genome segment encoding VP6 of Zambian G2P[4] sequences. The study strains are indicated with light-green circular symbols. Lineages are indicated in Roman numerals. The scale number indicates the number of nucleotide substitutions per site.

**Figure 7:**
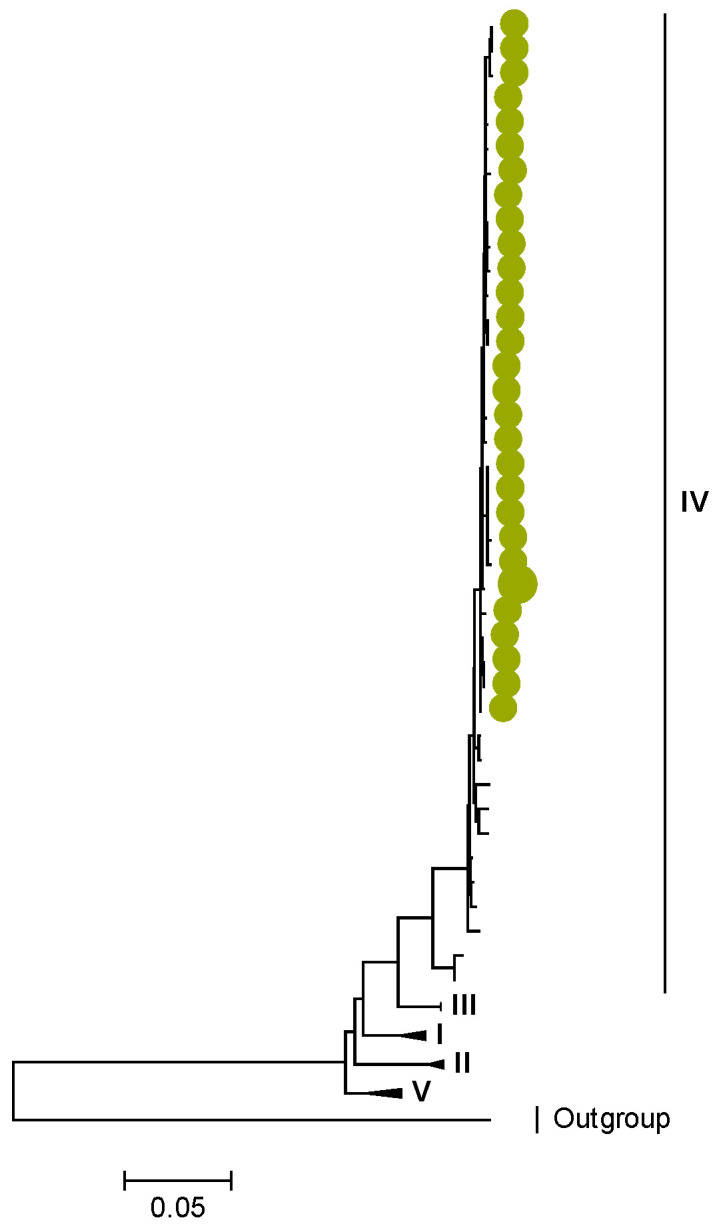
NSP1. Maximum Likelihood phylogenetic tree of the genome segment encoding NSP1 of Zambian G2P[4] sequences. The study strains are indicated with light-green circular symbols. Lineages are indicated in Roman numerals. The scale number indicates the number of nucleotide substitutions per site.

**Figure 8:**
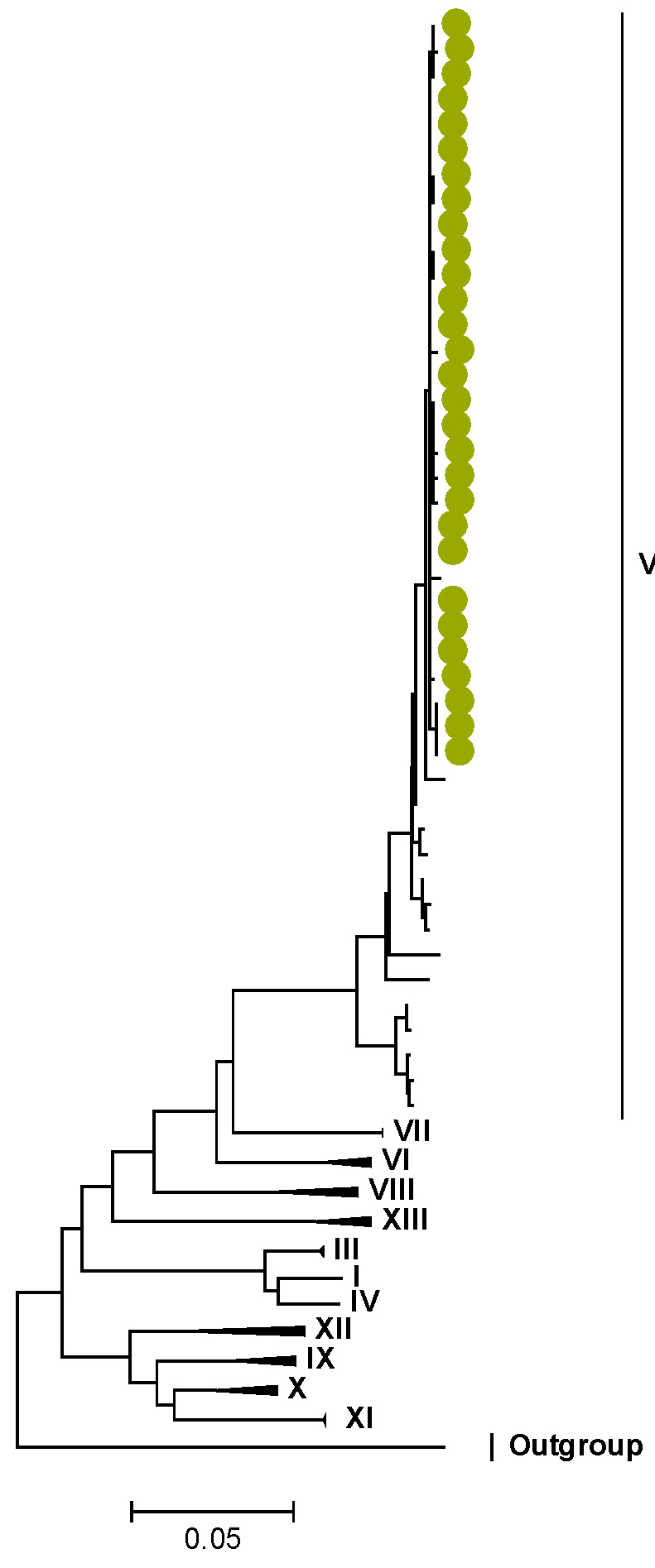
NSP2. Maximum Likelihood phylogenetic tree of the genome segment encoding NSP2 of Zambian G2P[4] sequences. The study strains are indicated with light-green circular symbols. Lineages are indicated in Roman numerals. The scale number indicates the number of nucleotide substitutions per site.

**Figure 9:**
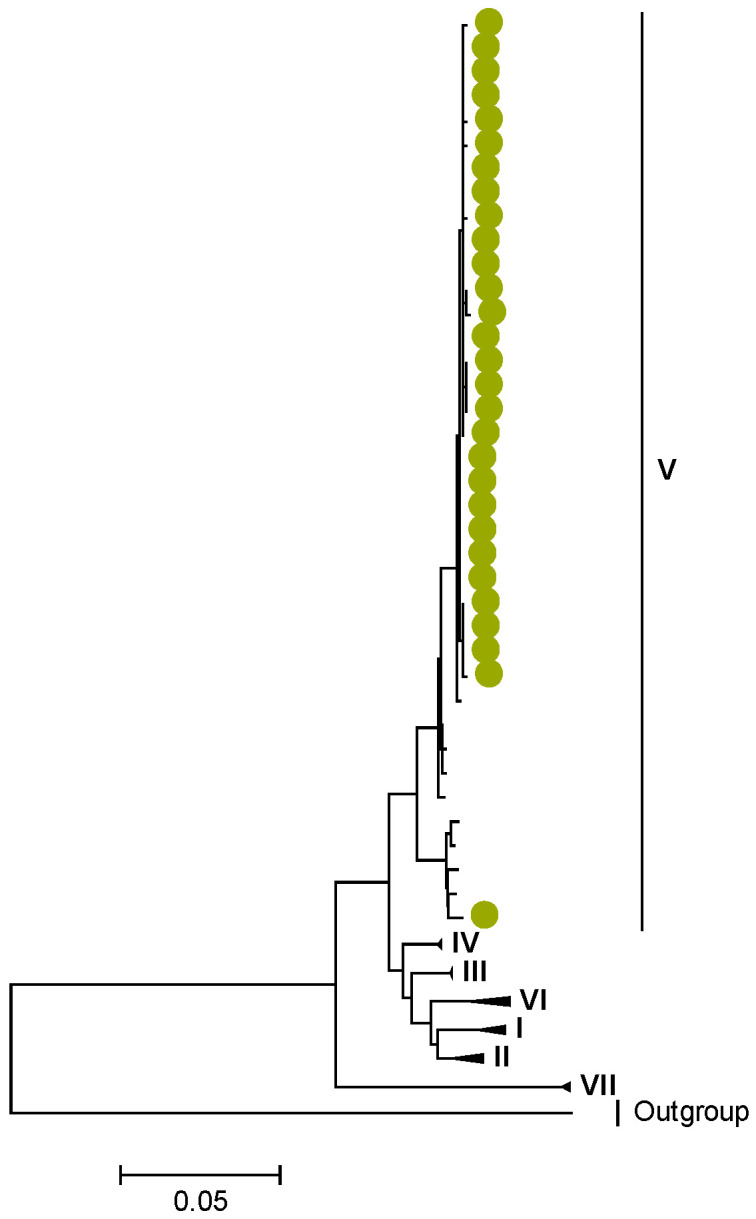
NSP3. Maximum Likelihood phylogenetic tree of the genome segment encoding NSP3 of Zambian G2P[4] sequences. The study strains are indicated with light-green circular symbols. Lineages are indicated in Roman numerals. The scale number indicates the number of nucleotide substitutions per site.

**Figure 10:**
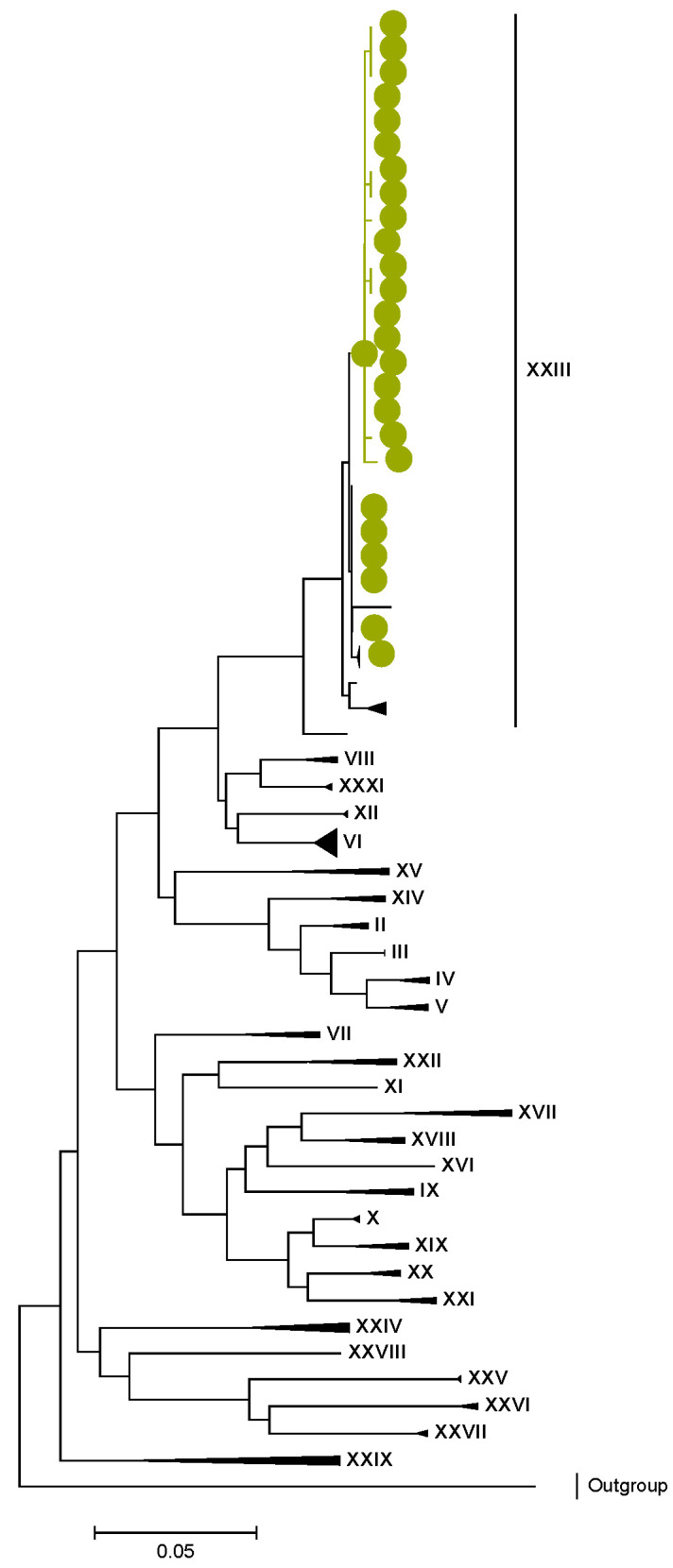
NSP4. Maximum Likelihood phylogenetic tree of the genome segment encoding NSP4 of Zambian G2P[4] sequences. The study strains are indicated with light-green circular symbols. Lineages are indicated in Roman numerals. The scale number indicates the number of nucleotide substitutions per site.

**Figure 11:**
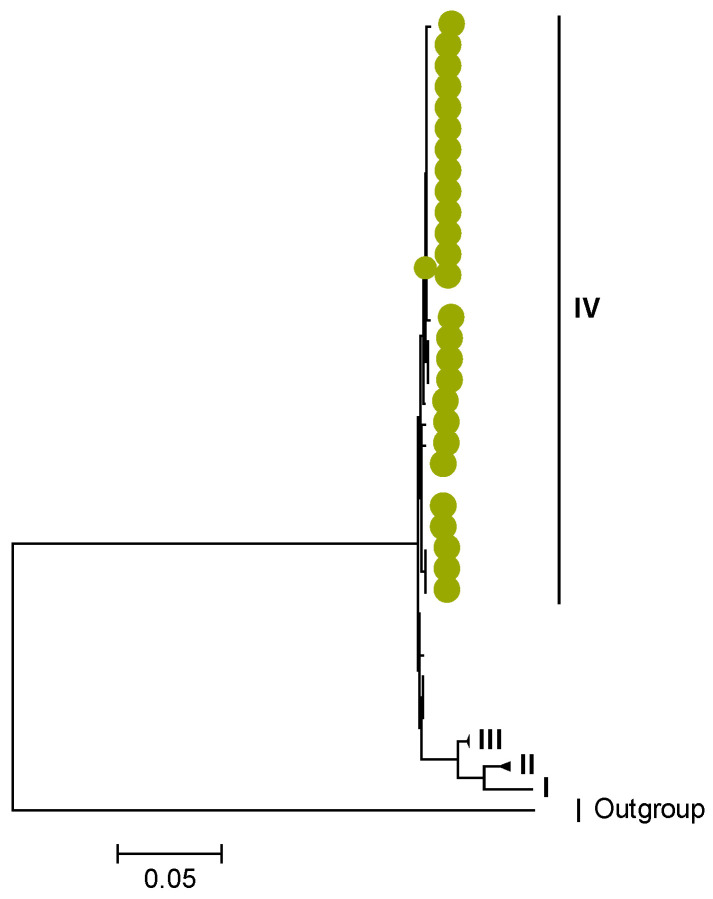
NSP5. Maximum Likelihood phylogenetic tree of the genome segment encoding NSP5 of Zambian G2P[4] sequences. The study strains are indicated with light-green circular symbols. Lineages are indicated in Roman numerals. The scale number indicates the number of nucleotide substitutions per site.

**Table 1:** Amino acid (AA) differences observed across the G2 lineages.

Lineages and Sub-Lineages	Representative G2 Sequences	AA and Position			
		44	96	178	287
**G2-Lineage I**	RVA/Human-tc/USA/DS-1/1976/G2P[4]	I	D	S	I
	RVA/Human-wt/TWN/TW6/1981/G2P[4]	I	D	S	I
**G2-Lineage II**	RVA/Human-tc/KEN/D205/1989/G2P[4]	I	D	S	I
	RVA/Human-wt/ITA/PAI11/1996/G2P[4]	I	D	S	I
**G2-Lineage III**	RVA/Human-wt/JPN/TMCII/1980/G2P[4]	I	D	S	I
	RVA/Human-wt/JPN/KUN/1980/G2P[4]	I	D	S	I
**G2-Lineage IV-*Sub-lineage IVa_1***	RVA/Human-wt/BRA/1250 06RJ/2006/G2P[4]	M	N	N	V
	RVA/Human-wt/THA/CU438-KK/09/2009/G2P[4]	M	N	N	V
**G2-Lineage IV-*Sub-lineage IVa_2***	RVA/Human-wt/ZAF/SA4476DB/97/1997/G2P[4]	M	N	N	V
	RVA/Human-wt/ZAF/SA4419SB/97/1997/G2P[4]	M	N	N	V
**G2-Lineage IV-*Sub-lineage IVa_3***	RVA/Human-wt/NPL/04N618/2004/G2P[4]	M	N	N	V
	RVA/Human-wt/NPL/04N618/2004/G2P[4]	M	N	N	V
	**RVA/Human-wt/ZMB/UFS-NGS-MRC-DPRU1724/2012/G2P[4]**	M	N	N	V
	**RVA/Human-wt/ZMB/UFS-NGS-MRC-DPRU1729/2012/G2P[4]**	M	N	N	V
	**RVA/Human-wt/ZMB/UFS-NGS-MRC-DPRU4691/2014/G2P[4]**	M	N	N	V
	**RVA/Human-wt/ZMB/UFS-NGS-MRC-DPRU4699/2014/G2P[4]**	M	N	N	V
	**RVA/Human-wt/ZMB/UFS-NGS-MRC-DPRU9556/2015/G2P[4]**	M	N	N	V
	**RVA/Human-wt/ZMB/UFS-NGS-MRC-DPRU9559/2015/G2P[4]**	M	N	N	V
	**RVA/Human-wt/ZMB/UFS-NGS-MRC-DPRU16639/2016/G2P[4]**	M	N	N	V
	**RVA/Human-wt/ZMB/UFS-NGS-MRC-DPRU16646/2016/G2P[4]**	M	N	N	V
**G2-Lineage V**	RVA/Human-wt/AUS/CK20055/2010/G2P[4]	I	D	S	I
	RVA/Human-wt/AUS/CK20037/2008/G2P[4]	I	D	S	I

The table summarizes the different AA differences observed between lineage IV G2 strains (highlighted in light brown), where Zambian G2 strains grouped, and representative strains of defined G2 lineages. The representative study strains are highlighted in bold-black.

**Table 2:** The AA properties of observed AA substitutions across G2 lineages and region of occurrence in VP7.

Amino Acid Substitution	Region	Amino Acid Property
**I44M**	Cytotoxic T lymphocyte	No change in charge or polarity
**D96N**	Neutralization epitope region 7-1a	Polar negatively charged to polar neutral residue
**S178N**	β-barrel domain	No change in charge or polarity
**I287V**	Rossmann-fold domain	

The table summarizes the amino acid differences observed between defined G2 lineages in respect to lineage IV where the study strains clustered, their region of occurrence in VP7, and the properties of the AA residues.

**Table 3:** Amino acid (AA) differences observed across the P[4] lineages.

Lineages	Representative P[4] Sequences	AA and Position		
		120	598	630
**P[4]-Lineage I**	RVA/Human-wt/AUS/CK20001/1977/G2P4	I	S	M
	RVA/Human-tc/USA/DS-1/1976/G2P4	I	S	M
**P[4]-Lineage II**	RVA/Human-wt/MWI/BID124/2012/G2P4	I	S	M
	RVA/Human-wt/ITA/PAI11/1996/G2P[4]	I	S	M
**P[4]-Lineage III**	RVA/Human-tc/JPN/KUN/1980/G2P4	I	S	M
	RVA/Human-wt/JPN/TMCII/1980/G2P[4]	I	S	M
**P[4]-Lineage IV**	RVA/Human-wt/UGA/MRC-DPRU3710/2009/G2P4	V	L	I
	RVA/Human-wt/MWI/BID19T/2012/G2P4	V	L	I
	**RVA/Human-wt/ZMB/UFS-NGS-MRC-DPRU1724/2012/G2P[4]**	V	L	I
	**RVA/Human-wt/ZMB/UFS-NGS-MRC-DPRU1729/2012/G2P[4]**	V	L	I
	**RVA/Human-wt/ZMB/UFS-NGS-MRC-DPRU4691/2014/G2P[4]**	V	L	I
	**RVA/Human-wt/ZMB/UFS-NGS-MRC-DPRU4699/2014/G2P[4]**	V	L	I
	**RVA/Human-wt/ZMB/UFS-NGS-MRC-DPRU9556/2015/G2P[4]**	V	L	I
	**RVA/Human-wt/ZMB/UFS-NGS-MRC-DPRU9559/2015/G2P[4]**	V	L	I
	**RVA/Human-wt/ZMB/UFS-NGS-MRC-DPRU16639/2016/G2P[4]**	V	L	I
	**RVA/Human-wt/ZMB/UFS-NGS-MRC-DPRU16646/2016/G2P[4]**	V	L	I

The table summarizes the different AA differences observed between lineage IV P[4] strains (highlighted in light brown), where Zambian P[4] strains grouped, and representative strains of defined P[4] lineages. The representative study strains are highlighted in bold-black.

**Table 4:** The AA properties of observed AA substitutions across P[4] lineages and region of occurrence in VP4.

Amino Acid Substitution	Region	Amino Acid Property
**I120V**	Hemagglutination domain	No change in charge or polarity
**S598L**	VP5 *	Polar neutral to nonpolar neutrally charged residue
**M630I**	VP5 *	No change in charge or polarity

The table summarizes the amino acid differences observed between defined P[4] lineages in respect to lineage IV where the study strains clustered, their region of occurrence in VP4, and the properties of the AA residues. The asterisk after “VP5” in indicates that it is a specific protein generated after trypsin cleavage of VP4.

## Data Availability

All the gene sequences in this study were submitted in the *NCBI GenBank* database under accession numbers OQ132993–OQ133311 and are included as supplementary data S4.

## Supplementary Materials

The following supporting information can be downloaded at: https://www.mdpi.com/article/10.3390/v15020501/s1, Supplementary Data S1: Accession numbers for the reference sequences used in the construction of the maximum likelihood phylogenetic trees; Supplementary Data S2: Nucleotide similarity analysis amongst genome segments of Zambian G2P[4] strains 3; Supplementary Data S3: Tables S1–S17.
