# Supplementary material for: Genomic Analysis of G2P[4] Group A Rotaviruses in Zambia Reveals Positive Selection in Amino Acid Site 7 of Viral Protein 3
Source: Viruses. 2023 Feb 11;15(2):501. doi: 10.3390/v15020501 (PMC9965253; doi:10.3390/v15020501)
Supplement: Supplementary file 1 [file viruses-15-00501-s001.zip › supplementary revised data S3.pdf]

Supplementary data S3: Supplementary tables S1-S17

Table S1: Amino acid differences observed across the R2 lineages.

Lineages	Representative R2 sequences	AA
		159
R2- Lineage I	RVA/Human-tc/USA/DS-1/1976/G2P[4]	K
	RVA/Human-wt/AUS/CK20001/1977/G2P[4]	K
R2-Lineage II	RVA/Human-tc/PHL/L26/1987/G12P[4]	K
	RVA/Human-tc/KEN/D205/1989/G2P[4]	K
R2-Lineage III	RVA/Human-tc/JPN/80SR001/1980/G2P[4]	K
	RVA/Human-tc/JPN/KUN/1980/G2P[4]	K
R2-Lineage IV	RVA/Human-tc/JPN/AU605/1986/G2P[4]	K
	RVA/Human-wt/CHN/TB-Chen/1996/G2P[4]	K
R2-Lineage V	RVA/Human-wt/AUS/D388/2013/G3P[8]	R
	RVA/Human-wt/ESP/SS98242319/2015/G3P[8]	R
	**RVA/Human-wt/ZMB/UFS-NGS-MRC-DPRU1724/2012/G2P[4]**	R
	**RVA/Human-wt/ZMB/UFS-NGS-MRC-DPRU1729/2012/G2P[4]**	R
	**RVA/Human-wt/ZMB/UFS-NGS-MRC-DPRU4691/2014/G2P[4]**	R
	**RVA/Human-wt/ZMB/UFS-NGS-MRC-DPRU4699/2014/G2P[4]**	R
	**RVA/Human-wt/ZMB/UFS-NGS-MRC-DPRU9556/2015/G2P[4]**	R
	**RVA/Human-wt/ZMB/UFS-NGS-MRC-DPRU9559/2015/G2P[4]**	R
	**RVA/Human-wt/ZMB/UFS-NGS-MRC-DPRU16639/2016/G2P[4]**	R
	**RVA/Human-wt/ZMB/UFS-NGS-MRC-DPRU16646/2016/G2P[4]**	R
R2-Lineage VI	RVA/Human-wt/BEL/B1711/2002/G6P[6]	K
	RVA/Human-wt/GHA/MRC-DPRU1818/1999/G2P[6]	K
R2-Lineage VII	RVA/Sheep-tc/CHN/CC0812-1/2008/G10P[15]	K
	RVA/Sheep-tc/CHN/LLR/1985/G10P[12]	K
R2-Lineage VIII	RVA/Human-tc/JPN/AU109/1994/G8P[4]	K
	RVA/Human-wt/HUN/BP[1]062/2004/G8P[14]	K
R2-Lineage IX	RVA/Human-tc/GBR/A64/1987/G10P[14]	K
	RVA/Human-tc/IND/69M/1980/G8P[4]	K
R2-Lineage X	RVA/Human-wt/ITA/PAH136/1996/G3P[9]	K
	RVA/Human-wt/HUN/Hun5/1997/G6P[14]	K
R2-Lineage XI	RVA/Human-wt/VNM/RVN1149/2014/G8P[8]	K
	RVA/Human-wt/JPN/UR14-10/2014/G8P[8]	K
R2-Lineage XII	RVA/Human-wt/HUN/ERN5471/2012/G2P[4]	K
	RVA/Human-wt/IND/N292/2004/G10P[11]	K
R2-Lineage XIII	RVA/Human-wt/TUN/17237/2008/G6P[9]	K
	RVA/Cow-tc/ZAF/O Agent/1965/G8P[1]	K
R2-Lineage XIV	RVA/Horse-wt/IRL/04V2024/2004/G14P[12]	K
	RVA/Horse-wt/ZAF/EqRV-SA1/2006/G14P[12]	K

The table summarizes the different AA differences observed between lineage V R2 strains (highlighted in light-brown), where Zambian R2 sequences grouped and representative strains of defined R2 lineages. The representative study strains are highlighted in bold-black.

Table S2: The AA properties of observed AA substitutions across R2 lineages and region of occurrence in VP[1].

Amino acid substitution	Region	Amino acid property
K159R	N-terminal domain	No change in charge or polarity;

The table summarizes the amino acid differences observed between defined R2 lineages in respect to lineage V where the study strains clustered, their region of occurrence in VP1 and the properties of the AA residues.

Table S3: Amino acid differences observed across the C2 lineages.

Lineages	Representative C2 sequences	AA and position		
		28	70	538
C2- Lineage I	RVA/Human-tc/USA/DS-1/1976/G2P[4]	N	I	V
	RVA/Human-wt/AUS/CK20001/1977/G2P[4]	N	I	V
C2-Lineage II	RVA/Human-tc/KEN/AK26/1982/G2P[4]	N	I	V
	RVA/Human-tc/KEN/D205/1989/G2P[4]	N	I	V
C2-Lineage III	RVA/Human-tc/JPN/80SR001/1980/G2P[4]	N	I	V
	RVA/Human-tc/JPN/KUN/1980/G2P[4]	N	I	V
C2-Lineage IV	RVA/Human-wt/AUS/CK20051/2010/G2P[4]	I	V	I
	**RVA/Human-wt/ZMB/UFS-NGS-MRC-DPRU1724/2012/G2P[4]**	I	V	I
	**RVA/Human-wt/ZMB/UFS-NGS-MRC-DPRU1729/2012/G2P[4]**	I	V	I
	**RVA/Human-wt/ZMB/UFS-NGS-MRC-DPRU4691/2014/G2P[4]**	I	V	I
	**RVA/Human-wt/ZMB/UFS-NGS-MRC-DPRU4699/2014/G2P[4]**	I	V	I
	**RVA/Human-wt/ZMB/UFS-NGS-MRC-DPRU9556/2015/G2P[4]**	I	V	I
	**RVA/Human-wt/ZMB/UFS-NGS-MRC-DPRU9559/2015/G2P[4]**	I	V	I
	**RVA/Human-wt/ZMB/UFS-NGS-MRC-DPRU16639/2016/G2P[4]**	I	V	I
	**RVA/Human-wt/ZMB/UFS-NGS-MRC-DPRU16646/2016/G2P[4]**	I	V	I
C2-Lineage V	RVA/Human-tc/USA/HCR3A/1984/G3P[3]	D	I	V
	RVA/Human-tc/ISR/Ro1845/1985/G3P[3]	D	I	V
C2-Lineage VI	RVA/Human-wt/GHA/Ghan-149/2008/G8P[6]	D	I	V
	RVA/Human-wt/GHA/Ghan-113/2008/G8P[6]	D	I	V
C2-Lineage VII	RVA/Cow-tc/CHN/DQ-75/2008/G10P[11]	D	I	V
	RVA/Human-wt/HUN/BP[1]062/2004/G8P[14]	D	I	V
C2-Lineage VIII	RVA/Sheep-tc/CHN/CC0812-1/2008/G10P[15]	D	I	V
	RVA/Sheep-tc/CHN/LLR/1985/G10P[12]	D	I	V
C2-Lineage IX	RVA/Cat-wt/ITA/BA222/2005/G3P[9]	D	I	V
	RVA/Human-wt/HUN/Hun5/1997/G6P[14]	D	I	V
C2-Lineage X	RVA/Horse-tc/JPN/Dai-10/2008/G24P[3]	D	I	V
	RVA/Human-tc/ISR/Ro8059/1995/G6P[1]	D	I	V
C2-Lineage XI	RVA/Human-tc/IND/69M/1980/G8P[4]10	D	I	V
	RVA/Human-wt/JPN/KF17/2010/G6P[9]	D	I	V
C2-Lineage XII	RVA/Human-tc/USA/Se584/1998/G6P[9]	D	I	V
	RVA/Human-wt/TUN/17237/2008/G6P[9]	D	I	V
C2-Lineage XIII	RVA/Horse-tc/UK/H2/1976/G3P[12]	D	I	V
	RVA/Horse-tc/USA/FI-14/1981/G3P[12]	D	I	V
C2-Lineage XIV	RVA/cow/ZAF/MRC-DPRU1604/2007/G6P[1]	D	I	V
	RVA/Human-tc/ITA/PA169/1988/G6P[14]	D	I	V

The table summarizes the different AA differences observed between lineage IV C2 strains (highlighted in light-brown), where Zambian C2 sequences grouped and representative strains of defined C2 lineages. The representative study strains are highlighted in bold-black.

Table S4: The AA properties of observed AA substitutions across C2 lineages and region of occurrence in VP2.

Amino acid substitution	Region	Amino acid property
N/D28I	N-terminal	Polar neutral to nonpolar neutral
I70V	N-terminal	No change in charge or polarity
V538I	N-terminal	No change in charge or polarity

The table summarizes the amino acid differences observed between defined C2 lineages in respect to lineage IV where the study strains clustered, their region of occurrence in VP2 and the properties of the AA residues.

Table S5: Amino acid differences observed across M2 lineages.

Lineages	Representative M2 sequences	AA and position		
		49	209	348
M2- Lineage I	RVA/Human-tc/USA/DS-1/1976/G2P[4]	S	V	M
M2-Lineage II	RVA/Human-tc/KEN/AK26/1982/G2P[4]	S	V	M
	RVA/Human-tc/KEN/D205/1989/G2P[4]	S	V	M
M2-Lineage III	RVA/Human-tc/JPN/80SR001/1980/G2P[4]	S	V	M
	RVA/Human-tc/JPN/KUN/1980/G2P[4]	S	V	M
M2-Lineage IV	RVA/Human-tc/CHN/TB-Chen/1996/G2P[4]	S	V	M
M2-Lineage V	RVA/Human-wt/MWI/BID11S/2012/G2P[4]	N	V	V
	RVA/Human-wt/BEL/BE83/2010/G2P[4]	N	V	V
	**RVA/Human-wt/ZMB/UFS-NGS-MRC-DPRU1724/2012/G2P[4]**	N	I	V
	**RVA/Human-wt/ZMB/UFS-NGS-MRC-DPRU1729/2012/G2P[4]**	N	I	V
	**RVA/Human-wt/ZMB/UFS-NGS-MRC-DPRU4691/2014/G2P[4]**	N	I	V
	**RVA/Human-wt/ZMB/UFS-NGS-MRC-DPRU4699/2014/G2P[4]**	N	I	V
	**RVA/Human-wt/ZMB/UFS-NGS-MRC-DPRU9556/2015/G2P[4]**	N	I	V
	**RVA/Human-wt/ZMB/UFS-NGS-MRC-DPRU9559/2015/G2P[4]**	N	I	V
	**RVA/Human-wt/ZMB/UFS-NGS-MRC-DPRU16639/2016/G2P[4]**	N	I	V
	**RVA/Human-wt/ZMB/UFS-NGS-MRC-DPRU16646/2016/G2P[4]**	N	I	V
M2-Lineage VI	RVA/Human-tc/SWE/1076/1983/G2P[6]	S	V	M
	RVA/Human-wt/GHA/Ghan-059/2008/G8P[1]	S	V	M
M2-Lineage VII	RVA/Human-wt/GHA/GHPML1989/2012/G2P[4]	S	V	M
	RVA/Goat-tc/BGD/GO34/1999/G6P[1]	S	V	T
C2-Lineage VIII	RVA/Human-wt/HUN/Hun5/1997/G6P[14]	S	V	T
C2-Lineage IX	RVA/Sheep-tc/CHN/LLR/1985/G10P[1]	S	V	M
	RVA/Sheep-tc/CHN/CC0812-1/2008/G10P[15]	S	V	M
C2-Lineage X	RVA/Human-tc/IND/69M/1980/G8P[4]	S	V	I
	RVA/Cow-tc/USA/NCDV/1967/G6P[6]1	S	V	A
C2-Lineage XI	RVA/Human-wt/BEL/B1711/2002/G6P[6]	S	V	I
	RVA/Human-wt/ITA/PAH136/1996/G3P[9]	S	V	I
C2-Lineage XII	RVA/Human-tc/KEN/D205/1989/G2P[4]	S	V	T
	RVA/Human-tc/MWI/MW1-131/1997/G8P[6]	S	V	I
C2-Lineage XIII	RVA/Human-wt/GHA/GH019-08/2008/G8P[6]	S	V	I
	RVA/Human-wt/GHA/GH018-08/2008/G8P[6]	S	V	I
C2-Lineage XIV	RVA/Cow-tc/ZAF/O Agent/1965/G8P[1]	S	V	I
	RVA/Human-XX/KEN/B12/1987/G8P[1]	S	V	I

The table summarizes the different AA differences observed between lineage V M2 strains (highlighted in light-brown), where Zambian M2 sequences grouped and representative strains of defined M2 lineages. The representative study strains are highlighted in bold-black.

Table S6: The AA properties of observed AA substitutions across M2 lineages and region of occurrence in VP3.

Amino acid substitution	Region	Amino acid property
S49N	N-terminal domain	No change in charge or polarity
V209I	Guanine-N7-Methyltransferase (N7-MTase) domain	No change in charge or polarity
M348V	2′-O-Methyltransfrase (2′-O-MTase) domain	No change in charge or polarity

The table summarizes the amino acid differences observed between defined M2 lineages in respect to lineage V where the study strains clustered, their region of occurrence in VP3 and the properties of the AA residues.

Table S7: Amino acid differences observed across NSP1 lineages.

Lineages	Representative A2 sequences	Amino acid			
		273	319	333	429
A2- Lineage I	RVA/Human-tc/USA/DS-1/1976/G2P[4]	L	H	W	K
	RVA/Human-wt/AUS/CK20001/1977/G2P[4]	L	H	W	K
A2-Lineage II	RVA/Human-wt/JPN/KN041/2012/G1P[8]	L	H	W	K
	RVA/Human-wt/DEU/GER1H-09/2009/G8P[4]	L	H	W	K
A2-Lineage III	RVA/Human-tc/JPN/80SR001/1980/G2P[4]	L	H	W	K
	RVA/Human-tc/JPN/KUN/1980/G2P[4]	L	H	W	K
A2-Lineage IV	RVA/Human-wt/MWI/BID11E/2012/G2P[4]	F	R	L	R
	**RVA/Human-wt/ZMB/UFS-NGS-MRC-DPRU1724/2012/G2P[4]**	F	R	L	R
	**RVA/Human-wt/ZMB/UFS-NGS-MRC-DPRU1729/2012/G2P[4]**	F	R	L	R
	**RVA/Human-wt/ZMB/UFS-NGS-MRC-DPRU4691/2014/G2P[4]**	F	R	L	R
	**RVA/Human-wt/ZMB/UFS-NGS-MRC-DPRU4699/2014/G2P[4]**	F	R	L	R
	**RVA/Human-wt/ZMB/UFS-NGS-MRC-DPRU9556/2015/G2P[4]**	F	R	L	R
	**RVA/Human-wt/ZMB/UFS-NGS-MRC-DPRU9559/2015/G2P[4]**	F	R	L	R
	**RVA/Human-wt/ZMB/UFS-NGS-MRC-DPRU16639/2016/G2P[4]**	F	R	L	R
	**RVA/Human-wt/ZMB/UFS-NGS-MRC-DPRU16646/2016/G2P[4]**	F	R	L	R
A2-Lineage V	RVA/Human-tc/SWE/1076/1983/G2P[6]	L	H	W	K
	RVA/Human-tc/PHI/L26/1987/G12P[4]	L	H	W	K

The table summarizes the different AA differences observed between lineage IV M2 strains (highlighted in light-brown), where Zambian A2 sequences grouped and representative strains of defined A2 lineages. The representative study strains are highlighted in bold-black.

Table S8: The AA properties of observed AA substitutions across M2 lineages and region of occurrence in NSP1.

Amino acid substitution	Region	AA property
L273F	Cytoskeleton-localization domain	No change in charge or polarity
H319R	IRF-binding domain	No change in charge or polarity
W333L	IRF-binding domain	No change in charge or polarity
K429R	IRF-binding domain	No change in charge or polarity

***Interferon Regulatory Factor (IRF)**

The table summarizes the amino acid differences observed between defined A2 lineages in respect to lineage IV where the study strains clustered, their region of occurrence in NSP1 and the properties of the AA residues.

Table S9: Amino acid differences observed across NSP2 lineages.

Lineages	Representative N2 sequences	AA 273
A2- Lineage I	RVA/Human-tc/USA/DS-1/1976/G2P[4]	L
	RVA/Human-wt/AUS/CK20001/1977/G2P[4]	L
A2-Lineage II	RVA/Human-wt/JPN/KN041/2012/G1P[8]	L
	RVA/Human-wt/DEU/GER1H-09/2009/G8P[4]	L
A2-Lineage III	RVA/Human-tc/JPN/80SR001/1980/G2P[4]	L
	RVA/Human-tc/JPN/KUN/1980/G2P[4]	L
A2-Lineage IV	RVA/Human-wt/MWI/BID11E/2012/G2P[4]	F
	**RVA/Human-wt/ZMB/UFS-NGS-MRC-DPRU1724/2012/G2P[4]**	F
	**RVA/Human-wt/ZMB/UFS-NGS-MRC-DPRU1729/2012/G2P[4]**	F
	**RVA/Human-wt/ZMB/UFS-NGS-MRC-DPRU4691/2014/G2P[4]**	F
	**RVA/Human-wt/ZMB/UFS-NGS-MRC-DPRU4699/2014/G2P[4]**	F
	**RVA/Human-wt/ZMB/UFS-NGS-MRC-DPRU9556/2015/G2P[4]**	F
	**RVA/Human-wt/ZMB/UFS-NGS-MRC-DPRU9559/2015/G2P[4]**	F
	**RVA/Human-wt/ZMB/UFS-NGS-MRC-DPRU16639/2016/G2P[4]**	F
	**RVA/Human-wt/ZMB/UFS-NGS-MRC-DPRU16646/2016/G2P[4]**	F
A2-Lineage V	RVA/Human-tc/SWE/1076/1983/G2P[6]	L
	RVA/Human-tc/PHI/L26/1987/G12P[4]	L

The table summarizes the different AA differences observed between lineage IV N2 strains (highlighted in light-brown), where Zambian N2 sequences grouped and representative strains of defined N2 lineages. The representative study strains are highlighted in bold-black.

Table S10: The AA properties of observed AA substitutions across N2 lineages and region of occurrence in NSP2.

Amino acid substitution	Region	Amino acid property
L23F	N terminal domain	No change in polarity or charge

The table summarizes the amino acid differences observed between defined N2 lineages in respect to lineage IV where the study strains clustered, their region of occurrence in NSP2 and the properties of the AA residues.

Table S11: Amino acid differences observed across NSP3 lineages.

Lineages	Representative T2 sequences	AA
		61
T2- Lineage I	RVA/Human-tc/USA/DS-1/1976/G2P[4]	M
	RVA/Human-wt/AUS/CK20001/1977/G2P[4]	M
T2-Lineage II	RVA/Human-tc/KEN/AK26/1982/G2P[4]	M
	RVA/Human-tc/PHI/L26/1987/G12P[4]	M
T2-Lineage III	RVA/Human-tc/JPN/80SR001/1980/G2P[4]	M
	RVA/Human-tc/JPN/KUN/1980/G2P[4]	M
T2-Lineage IV	RVA/Human-wt/JPN/85A21410/1985/G2P[4]	M
	RVA/Human-tc/JPN/AU605/1986/G2P[4]	M
T2-Lineage V	RVA/Human-wt/MWI/BID1CT/2012/G2P[4]	I
	**RVA/Human-wt/ZMB/UFS-NGS-MRC-DPRU1724/2012/G2P[4]**	I
	**RVA/Human-wt/ZMB/UFS-NGS-MRC-DPRU1729/2012/G2P[4]**	I
	**RVA/Human-wt/ZMB/UFS-NGS-MRC-DPRU4691/2014/G2P[4]**	I
	**RVA/Human-wt/ZMB/UFS-NGS-MRC-DPRU4699/2014/G2P[4]**	I
	**RVA/Human-wt/ZMB/UFS-NGS-MRC-DPRU9556/2015/G2P[4]**	I
	**RVA/Human-wt/ZMB/UFS-NGS-MRC-DPRU9559/2015/G2P[4]**	I
	**RVA/Human-wt/ZMB/UFS-NGS-MRC-DPRU16639/2016/G2P[4]**	I
	**RVA/Human-wt/ZMB/UFS-NGS-MRC-DPRU16646/2016/G2P[4]**	I
T2-Lineage VI	RVA/Human-tc/SWE/1076/1983/G2P[6]	M
	RVA/Human-tc/PHI/L26/1987/G12P[4]	M
T2-Lineage VII	RVA/Human-tc/IND/69M/1980/G8P[4]	M
	RVA/Human-tc/IDN/57M/1980/G4P[1]	M

The table summarizes the different AA differences observed between lineage V T2 strains (highlighted in light-brown), where Zambian T2 sequences grouped and representative strains of defined T2 lineages. The representative study strains are highlighted in bold-black.

Table S12: The AA properties of observed AA substitutions across T2 lineages and region of occurrence in NSP3.

Amino acid substitution	Region	Amino acid property
M61I	C terminal domain	No change in polarity or charge

The table summarizes the amino acid differences observed between defined T2 lineages in respect to lineage V where the study strains clustered, their region of occurrence in NSP3 and the properties of the AA residues.

Table S13: Amino acid differences observed across the NSP4 lineages.

Lineages	Representative E2 sequences	AA
		137
E2- Lineage I	RVA/Human-tc/USA/DS-1/1976/G2P[4]	R
E2-Lineage II	RVA/Human-wt/ITA/PAI11/1996/G2P[4]	R
E2-Lineage III	RVA/Human-tc/JPN/80SR001/1980/G2P[4]	R
E2-Lineage IV	RVA/Human-tc/JPN/AU605/1986/G2P[4]	R
E2-Lineage V	RVA/Human-wt/JPN/MMC88/2005/G2P[4]	R
E2-Lineage VI	RVA/human-wt/JPN/OH3493/2012/G1P[8]	Q
E2-Lineage VII	RVA/Human-tc/EGY/AS997/2012/G9P[14]	R
E2-Lineage VIII	RVA/Human-tc/MWI/MAL39/2007/G12P[6]	R
E2-Lineage IX	RVA/Human-wt/GHA/Ghan-059/2008/G8P[1]	R
E2-Lineage X	RVA/Human-wt/CMR/ES293/2011/G3P[6]	R
E2-Lineage XI	RVA/Human-wt/AUS/D388/2013/G3P	R
E2-Lineage XII	RVA/Human-wt/JPN/MU14-3/2014/G8P[8]	Q
E2-Lineage XIII	RVA/Cow-wt/IND/RUBV189/XXXX/G3P[3]	Q
E2-Lineage XIV	RVA/Human-tc/KEN/D205/1989/G2P[4]	Q
E2-Lineage XV	RVA/Human-wt/COD/DRC88/2003/G8P[8]	R
E2-Lineage XVI	RVA/Human-tc/ITA/PA169/1988/G6P[14]	R
E2-Lineage XVII	RVA/Cat-wt/ITA/BA222/2005/G3P[9]	R
E2-Lineage XVIII	RVA/roe deer-wt/SLO/D110-15/2015/G8P[14]	R
E2-Lineage XIX	RVA/Camel-wt/SDN/MRC-DPRU447/2002/G8P[11]	R
E2-Lineage XX	RVA/Dog-wt/GER/88977/2013/G8P[1]	R
E2-Lineage XXI	RVA/Human-wt/GHA/GH019-08/2008/G8P[6]	R
E2-Lineage XXII	RVA/Human-wt/KEN/Keny-061/2008/G9P[6]	R
	RVA/Human-wt/MWI/BID1CT/2012/G2P[4]	Q
	RVA/Human-wt/KEN/KLF0630/2013/G2P[4]	Q
	**RVA/Human-wt/ZMB/UFS-NGS-MRC-DPRU1724/2012/G2P[4]**	Q
	**RVA/Human-wt/ZMB/UFS-NGS-MRC-DPRU1729/2012/G2P[4]**	Q
	**RVA/Human-wt/ZMB/UFS-NGS-MRC-DPRU4691/2014/G2P[4]**	Q
	**RVA/Human-wt/ZMB/UFS-NGS-MRC-DPRU4699/2014/G2P[4]**	Q
	**RVA/Human-wt/ZMB/UFS-NGS-MRC-DPRU9556/2015/G2P[4]**	Q
	**RVA/Human-wt/ZMB/UFS-NGS-MRC-DPRU9559/2015/G2P[4]**	Q
	**RVA/Human-wt/ZMB/UFS-NGS-MRC-DPRU16639/2016/G2P[4]**	Q
	**RVA/Human-wt/ZMB/UFS-NGS-MRC-DPRU16646/2016/G2P[4]**	Q
E2-Lineage XXIV	RVA/Cow-wt/DEN/DK11331/2007/GXP[X]	R
E2-Lineage XXV	RVA/Lamb-wt/CHN/LLR/1985/G10P[12]	R
E2-Lineage XXVI	RVA/Human-XX/USA/CC425/XXXX/G3P[9]	R
E2-Lineage XXVII	RVA/Horse-wt/TUR/Eskisehir/2011/G3P[12]	Q
E2-Lineage XXVIII	RVA/Horse-wt/JPN/J/1998/G3P[12]	R
E2-Lineage XXIX	RVA/Cow-wt/CHN/DQ-75/2008/G10P[11]	R
E2-Lineage XXXI	RVA/Human-tc/MWI/OP2-384/2001/G8P[6]	Q

The table summarizes the different AA differences observed between lineage XXII T2 strains (highlighted in light-brown), where Zambian E2 sequences grouped and representative strains of defined E2 lineages. The representative study strains are highlighted in bold-black.

Table S14: The AA properties of observed AA substitutions across E2 lineages and region of occurrence in NSP4.

Amino acid substitution	Region	Amino acid property
R137Q	Signal transducing domain	Polar positive to polar neutral

The table summarizes the amino acid differences observed between defined E2 lineages in respect to lineage XXII where the study strains clustered, their region of occurrence in NSP4 and the properties of the AA residues.

Table S15: Amino acid differences observed across NSP5 lineages.

Lineages	Representative H2 sequences	AA
		122
H2- Lineage I	RVA/Human-tc/USA/DS-1/1976/G2P[4]	V
	RVA/Human-wt/AUS/CK20001/1977/G2P[4]	V
H2-Lineage II	RVA/Human-wt/BRA/HSP034/1998/G2P[6]	V
	RVA/Human-wt/ITA/PAI11/1996/G2P[4]	V
H2-Lineage III	RVA/Human-tc/JPN/KUN/1980/G2P[4]	V
	RVA/Human-tc/JPN/80SR001/1980/G2P[4]	V
H2-Lineage IV	RVA/Human-wt/KEN/4054/2017/G2P[4]	M
	RVA/Human-wt/AUS/CK20051/2010/G2P[4]	M
	RVA/Human-wt/BRA/IP-47 MG/2008/G2P[4]	M
	RVA/Human-wt/BGN/J300/2010/G2P[4]	M
	RVA/Human-wt/USA/VU10-11-19/2011/G2P[4]	M
	RVA/Human-wt/AUS/WAPC703/2010/G2P[4]	M
	**RVA/Human-wt/ZMB/UFS-NGS-MRC-DPRU1724/2012/G2P[4]**	M
	**RVA/Human-wt/ZMB/UFS-NGS-MRC-DPRU1729/2012/G2P[4]**	M
	**RVA/Human-wt/ZMB/UFS-NGS-MRC-DPRU4691/2014/G2P[4]**	M
	**RVA/Human-wt/ZMB/UFS-NGS-MRC-DPRU4699/2014/G2P[4]**	M
	**RVA/Human-wt/ZMB/UFS-NGS-MRC-DPRU9556/2015/G2P[4]**	M
	**RVA/Human-wt/ZMB/UFS-NGS-MRC-DPRU9559/2015/G2P[4]**	M
	**RVA/Human-wt/ZMB/UFS-NGS-MRC-DPRU16639/2016/G2P[4]**	M
	**RVA/Human-wt/ZMB/UFS-NGS-MRC-DPRU16646/2016/G2P[4]**	M

The table summarizes the different AA differences observed between lineage IV H2 strains (highlighted in light-brown), where Zambian H2 sequences grouped and representative strains of defined H2 lineages. The representative study strains are highlighted in bold-black.

Table S16: The AA properties of observed AA substitutions across E2 lineages and region of occurrence in NSP5.

AA difference	Region	AA property
V122M	N terminal domain	No change in charge or polarity

The table summarizes the amino acid differences observed between defined H2 lineages in respect to lineage IV where the study strains clustered, their region of occurrence in NSP5 and the properties of the AA residues.

Table S17: Positively selected amino acid sites in 29 Zambian G2P[4] rotavirus sequences as identified by FEL, FUBAR, and MEME analysis.

Genome segment	Positively selected sites as analysed by FEL, FUBAR and MEME		
	FEL	FUBAR	MEME
**VP1**	-	-	703, 969
**VP2**	-	-	-
**VP3**	7*	7*	7*
**VP4**	-	-	-
**VP6**			
**VP7**	-	-	-
**NSP1**	-	480	-
**NSP2**	-	-	-
**NSP3**	-	-	-
**NSP4**	-	-	-
**NSP5**	-	-	-

Positively selected amino acid sites are indicated by asterisk sign. The dash (-) sign indicates no positively site was identified.

Table S18: The two different AA residues identified at VP3 site 7 of Zambian G2P[4] sequences.

Zambian G2P[4] VP3 sequences	AA site 7
RV A/Human-wt/ZMB/UFS-NGS-MRC-DPRU1724/2012/G2P[4]	I
RV A/Human-wt/ZMB/UFS-NGS-MRC-DPRU1729/2012/G2P[4]	I
RV A/Human-wt/ZMB/UFS-NGS-MRC-DPRU4691/2014/G2P[4]	I
RV A/Human-wt/ZMB/UFS-NGS-MRC-DPRU4699/2014/G2P[4]	R
RV A/Human-wt/ZMB/UFS-NGS-MRC-DPRU4705/2014/G2P[4]	R
RV A/Human-wt/ZMB/UFS-NGS-MRC-DPRU4714/2014/G2P[4]	R
RV A/Human-wt/ZMB/UFS-NGS-MRC-DPRU4718/2014/G2P[4]	R
RV A/Human-wt/ZMB/UFS-NGS-MRC-DPRU4736/2014/G2P[4]	R
RV A/Human-wt/ZMB/UFS-NGS-MRC-DPRU4770/2014/G2P[4]	R
RV A/Human-wt/ZMB/UFS-NGS-MRC-DPRU4819/2014/G2P[4]	R
RV A/Human-wt/ZMB/UFS-NGS-MRC-DPRU9393/2015/G2P[4]	R
RV A/Human-wt/ZMB/UFS-NGS-MRC-DPRU9420/2015/G2P[4]	R
RV A/Human-wt/ZMB/UFS-NGS-MRC-DPRU9438/2015/G2P[4]	R
RV A/Human-wt/ZMB/UFS-NGS-MRC-DPRU9464/2015/G2P[4]	I
RV A/Human-wt/ZMB/UFS-NGS-MRC-DPRU9475/2015/G2P[4]	I
RV A/Human-wt/ZMB/UFS-NGS-MRC-DPRU9495/2015/G2P[4]	I
RV A/Human-wt/ZMB/UFS-NGS-MRC-DPRU9502/2015/G2P[4]	R
RV A/Human-wt/ZMB/UFS-NGS-MRC-DPRU9503/2015/G2P[4]	R
RV A/Human-wt/ZMB/UFS-NGS-MRC-DPRU9515/2015/G2P[4]	I
RV A/Human-wt/ZMB/UFS-NGS-MRC-DPRU9556/2015/G2P[4]	R
RV A/Human-wt/ZMB/UFS-NGS-MRC-DPRU9559/2015/G2P[4]	I
RV A/Human-wt/ZMB/UFS-NGS-MRC-DPRU13231/2016/G2P[4]	I
RV A/Human-wt/ZMB/UFS-NGS-MRC-DPRU13301/2016/G2P[4]	I
RV A/Human-wt/ZMB/UFS-NGS-MRC-DPRU13352/2016/G2P[4]	R
RV A/Human-wt/ZMB/UFS-NGS-MRC-DPRU13545/2016/G2P[4]	R
RV A/Human-wt/ZMB/UFS-NGS-MRC-DPRU16599/2016/G2P[4]	R
RV A/Human-wt/ZMB/UFS-NGS-MRC-DPRU16601/2016/G2P[4]	I
RV A/Human-wt/ZMB/UFS-NGS-MRC-DPRU16639/2016/G2P[4]	R
RV A/Human-wt/ZMB/UFS-NGS-MRC-DPRU16646/2016/G2P[4]	R

The table displays the two AA residues, arginine and isoleucine that were identified in AA site 7 of Zambian VP3 sequences. Arginine was identified in 18 of the sequences while 11 featured an isoleucine residue.
