# Supplementary material for: Genomic Analysis of G2P[4] Group A Rotaviruses in Zambia Reveals Positive Selection in Amino Acid Site 7 of Viral Protein 3
Source: Viruses. 2023 Feb 11;15(2):501. doi: 10.3390/v15020501 (PMC9965253; doi:10.3390/v15020501)
Supplement: Supplementary file 1 [file viruses-15-00501-s001.zip › Supplementary_data S1.pdf]

Supplementary data S1: Accession numbers for the reference sequences used in the construction of the Maximum Likelihood phylogenetic trees.

VP7	VP4	VP6	VP1	VP2	VP3	NSP1	NSP2	NSP3	NSP4	NSP5
**Outgroup**	**Outgroup**	**Outgroup**	**Outgroup**	**Outgroup**	**Outgroup**	**Outgroup**	**Outgroup**	**Outgroup**	**Outgroup**	**Outgroup**
GU723327	JX406750	JX406752	JX406747	JX406748	JX406749	JX406751	JX406754	JX406753	JX406756	JX406757
**Lineage I**	**Lineage I**	**Lineage I**	**Lineage I**	**Lineage I**	**Lineage I**	**Lineage I**	**Lineage I**	**Lineage I**	**Lineage I**	**Lineage I**
EF672581	LC001901	DQ870507	DQ870505	HQ650117	HQ650118	HQ650120	HQ650123	HQ650122	HQ650125	HQ650126
AF044338	KC443590	HQ650121	KC443587		KC443589	KC443582		KC443584		KC443586
	HQ650119	KC443591								
**Lineage II**	**Lineage II**	**Lineage II**	**Lineage II**	**Lineage II**	**Lineage II**	**Lineage II**	**Lineage II**	**Lineage II**	**Lineage II**	**Lineage II**
JF304920	KJ752665	JF304930	DQ146693	JF304916	EF583035	AB975785	EU708908	JF304934	DQ525190	KC178755
	KJ752461		JF304915	JF304927	JF304928	GQ414546	EU708930	EF672593	KC178746	JQ715677
	KF636314						EU708952			
	MG892018									
	MG926728									
**Lineage III**	**Lineage III**	**Lineage III**	**Lineage III**	**Lineage III**	**Lineage III**	**Lineage III**	**Lineage III**	**Lineage III**	**Lineage III**	**Lineage III**
D50127	AB733131	AB733132	LC438390	AB733134	LC438392	AB733136	AB733137	AB733138	D88829	AB091727
D50124			AB733133	LC438391	AB733135	LC438396	LC438397		LC438399	LC438400
**Lineage IV**	**Lineage IV**	**Lineage IV**	**Lineage IV**	**Lineage IV**	**Lineage IV**	**Lineage IV**	**Lineage IV**	**Lineage IV**	**Lineage IV**	**Lineage IV**
	MG181837	AB762771	AB762772	KC443785	AB762774	KJ751619	AY787648	LC002076	AB762778	LC065027
	MG652350	AY787645	AY787653	EF554083	AY787654	EF554088		AB762777	AY87650	AB971565
	JQ069668			AY787652		AB762775				MH301253
	KP882275			LC065021		MG181827				KC443783
	KC443159					JX965145				JX965157
	KC443258					MT767401				KF716441
	KP007171					KC442887				KU248393
	KP753181					KU248378				
						KJ753518				
**Sub-lineage IVa-1**		**Lineage V**	**Lineage V**	**Lineage V**	**Lineage V**	**Lineage V**	**Lineage V**	**Lineage V**	**Lineage V**	
MH291294		EF554086	KU059766	EU708891	LC026108	JX416214	KU059773	LC074739	HM467979	
LC105588			KU550262	EU708902	KC443786	EF672592	LC002074.1	EF554090	HQ641371	
KC443789					JX965139		KC442959	MG181928		
MG181914					KC443786		KR705329	KJ752125		
					KC443622		MN223989	KC571499		
					KF716382		JX965148	KU361002		
					KF716381		KU059773	KP882535		
					LC060819		KJ753821	KP188805		
					KR705214		LC002074	MF184795		
					LC026108		KJ753486	KJ752615		
					KR705401		KM660141			

				MG181768	KJ583192		
				KJ752996			
**Sub-lineage IVa-2**	**Lineage VI**	**Lineage VI**	**Lineage VI**	**Lineage VI**		**Lineage VI**	**Lineage VI**
AY261354	KJ748480	EF554082	KP882630	JX416213		JX416216	AB796456
AY261355	KP882628	KJ751624	KP882685	KP882477		AB753500	LC228324
AY261353							
**Sub-lineage IVa-3**	**Lineage VII**	**Lineage VII**	**Lineage VII**	**Lineage VII**		**Lineage VII**	**Lineage VII**
MG181320	LC095956	FJ031024	FN665689	GU937879		EF672558	KX265688
MN066787	LC169856	JQ013502	GU384191	LC105582		JQ863317	AB975935
LC086796							
MZ094197							
KC443789							
JX965171							
JQ069505							
KP752665							
KU356590							
	**Lineage VIII**	**Lineage VIII**	**Lineage VIII**	**Lineage VIII**			**Lineage VIII**
	EF583020	LC065020	HQ834198	EF554106			AB938299
		FN665688	JQ013503				GU937886
	**Lineage IX**	**Lineage IX**	**Lineage IX**	**Lineage IX**			**Lineage IX**
	KP882925	EF583017	GU827407	JQ013504			LC119113
	EF554152	EF576937	EF554105	HQ834207			KP882481
	**Lineage X**	**Lineage X**	**Lineage X**	**Lineage X**			**Lineage X**
	DQ870496	GU296420	AB748581	EF583015			KM660203
	EF554130	EF554104	KC815659	DQ70495			KM660192
	**Lineage XI**	**Lineage XI**	**Lineage XI**	**Lineage XI**			**Lineage XI**
	EF583016	LCO7473	EF583014	EF554084			KUO59775
	KC175098	LC102997	JF421976	GU296424			
	Lineage XII	Lineage XII	Lineage XII	Lineage XII			Lineage XII
	AY740737	KJ919361	JX271002	JF304917			LC074740
	KP258402	KC175269	EF583042	AB751564			LC102992
	Lineage XIII	Lineage XIII	Lineage XIII	Lineage XIII			Lineage XIII
	AB573073	JX271001	KM454482	KJ748478			EF592592
	DQ838647	JF693037	KM454493	KJ748467			
	Lineage XIV	Lineage XIV	Lineage XIV				Lineage XIV
	EF554108	JN903527	EF554127				JF304924
	GU827410	JQ345489	KF636257				AB755562
	Lineage XV						Lineage XV
	JQ345494						GU25961
	AB908927						DQ005105
							Lineage XVI

EF554135
Lineage XVII
GU827415
GU296416
Lineage XVIII
KY426806
JN831207
Lineage XIX
KC257089
KP198645
Lineage XX
EF554157
KJ940160
Lineage XXI
KJ870898
KJ748485
Lineage XXII
EF672561
KP941135
Lineage XXIII
MG181621
MG181929
Lineage XXIV
JN248454
KP752503
LC055556
Lineage XXV
FJ031022
JQ031148
Lineage XXVI
AJ311728
AJ311729
Lineage XXVII
JQ687223
JN903510
Lineage XXVIII
KC815689
Lineage XXIX
GU181282
Lineage XXX
KY972050
KY972002
