# Supplementary material for: Genomic Analysis of G2P[4] Group A Rotaviruses in Zambia Reveals Positive Selection in Amino Acid Site 7 of Viral Protein 3
Source: Viruses. 2023 Feb 11;15(2):501. doi: 10.3390/v15020501 (PMC9965253; doi:10.3390/v15020501)
Supplement: Supplementary file 1 [file viruses-15-00501-s001.zip › Supplementary_data S2.pdf]

Supplementary data S2: Nucleotide similarities analysis amongst gene segments of Zambian G2P[4] strains.

VP1

		1	2	3	4	5	6	7	8	9	10	11	12	13	14	15	16	17	18	19	20	21	22	23	24	25	26	27	28	29
1	RVA/Human-wt/ZMB/UFS-NGS-MRC-DPRU1724/2012/G2P4																													
2	RVA/Human-wt/ZMB/UFS-NGS-MRC-DPRU1729/2012/G2P4	99.9																												
3	RVA/Human-wt/ZMB/UFS-NGS-MRC-DPRU4691/2014/G2P4	99.8	99.8																											
4	RVA/Human-wt/ZMB/UFS-NGS-MRC-DPRU4699/2014/G2P4	99.9	99.8	99.7																										
5	RVA/Human-wt/ZMB/UFS-NGS-MRC-DPRU4705/2014/G2P4	99.7	99.7	100.0	99.7																									
6	RVA/Human-wt/ZMB/UFS-NGS-MRC-DPRU4714/2014/G2P4	99.7	99.7	100.0	99.7	99.9																								
7	RVA/Human-wt/ZMB/UFS-NGS-MRC-DPRU4718/2014/G2P4	98.9	98.9	99.1	98.9	99.1	99.1																							
8	RVA/Human-wt/ZMB/UFS-NGS-MRC-DPRU4736/2014/G2P4	99.9	99.8	99.7	100.0	99.7	99.7	98.8																						
9	RVA/Human-wt/ZMB/UFS-NGS-MRC-DPRU4770/2014/G2P4	99.8	99.7	99.6	99.9	99.6	99.6	98.7	99.8																					
10	RVA/Human-wt/ZMB/UFS-NGS-MRC-DPRU4819/2014/G2P4	99.7	99.7	99.9	99.6	99.9	99.9	99.0	99.6	99.5																				
11	RVA/Human-wt/ZMB/UFS-NGS-MRC-DPRU9393/2015/G2P4	99.1	99.0	98.9	99.0	98.9	98.9	98.0	99.0	98.9	98.8																			
12	RVA/Human-wt/ZMB/UFS-NGS-MRC-DPRU9420/2015/G2P4	99.8	99.8	99.7	99.8	99.6	99.6	98.8	99.7	99.7	99.6	98.9																		
13	RVA/Human-wt/ZMB/UFS-NGS-MRC-DPRU9438/2015/G2P4	99.1	99.0	98.9	99.0	98.9	98.9	98.0	99.0	98.9	98.8	100.0	98.9																	
14	RVA/Human-wt/ZMB/UFS-NGS-MRC-DPRU9464/2015/G2P4	99.8	99.8	99.7	99.8	99.6	99.6	98.8	99.7	99.7	99.6	98.9	99.9	98.9																
15	RVA/Human-wt/ZMB/UFS-NGS-MRC-DPRU9475/2015/G2P4	99.1	99.0	98.9	99.0	98.9	98.9	98.0	99.0	98.9	98.8	100.0	98.9	100.0	98.9															
16	RVA/Human-wt/ZMB/UFS-NGS-MRC-DPRU9495/2015/G2P4	99.1	99.0	98.9	99.0	98.9	98.9	98.0	99.0	98.9	98.8	100.0	98.9	100.0	98.9	100.0														
17	RVA/Human-wt/ZMB/UFS-NGS-MRC-DPRU9502/2015/G2P4	99.8	99.8	99.7	99.8	99.7	99.7	98.8	99.8	99.7	99.7	98.9	99.9	98.9	99.9	98.9	98.9													
18	RVA/Human-wt/ZMB/UFS-NGS-MRC-DPRU9503/2015/G2P4	99.8	99.8	99.7	99.8	99.6	99.6	98.8	99.7	99.7	99.6	98.9	99.9	98.9	99.9	98.9	98.9	99.9												
19	RVA/Human-wt/ZMB/UFS-NGS-MRC-DPRU9515/2015/G2P4	99.8	99.8	99.6	99.7	99.6	99.6	98.8	99.7	99.7	99.6	98.9	99.9	98.9	99.9	98.9	98.9	99.9	99.8											
20	RVA/Human-wt/ZMB/UFS-NGS-MRC-DPRU9556/2015/G2P4	99.8	99.8	99.7	99.8	99.7	99.7	98.8	99.8	99.7	99.7	98.9	99.9	98.9	99.9	98.9	98.9	99.9	99.9	99.9										
21	RVA/Human-wt/ZMB/UFS-NGS-MRC-DPRU9559/2015/G2P4	99.8	99.8	99.7	99.8	99.6	99.6	98.8	99.7	99.7	99.6	98.9	99.8	98.9	99.8	98.9	98.9	99.8	99.8	99.8	99.8									
22	RVA/Human-wt/ZMB/UFS-NGS-MRC-DPRU13231/2016/G2P4	99.8	99.8	99.6	99.7	99.6	99.6	98.8	99.7	99.7	99.6	98.9	99.8	98.9	99.8	98.9	98.9	99.9	99.8	99.8	99.9	99.8								
23	RVA/Human-wt/ZMB/UFS-NGS-MRC-DPRU13301/2016/G2P4	97.5	97.5	97.5	97.6	97.4	97.4	96.6	97.6	97.5	97.4	97.5	97.4	97.5	97.4	97.5	97.5	97.5	97.4	97.4	97.5	97.4	97.4							
24	RVA/Human-wt/ZMB/UFS-NGS-MRC-DPRU13352/2016/G2P4	99.8	99.8	99.6	99.7	99.6	99.6	98.8	99.7	99.7	99.6	98.9	99.8	98.9	99.8	98.9	98.9	99.9	99.8	99.8	99.9	99.8	99.8	97.4						
25	RVA/Human-wt/ZMB/UFS-NGS-MRC-DPRU13545/2016/G2P4	99.8	99.8	99.7	99.8	99.6	99.6	98.8	99.7	99.7	99.6	98.9	99.9	98.9	99.9	98.9	98.9	99.9	99.9	99.8	99.9	99.8	100.0	97.4	99.8					
26	RVA/Human-wt/ZMB/UFS-NGS-MRC-DPRU16599/2016/G2P4	99.7	99.7	99.6	99.7	99.6	99.6	98.8	99.6	99.6	99.6	98.8	99.8	98.8	99.8	98.8	98.8	99.8	99.8	99.8	99.8	99.7	99.8	97.5	99.8	99.8				
27	RVA/Human-wt/ZMB/UFS-NGS-MRC-DPRU16601/2016/G2P4	99.7	99.7	99.6	99.7	99.6	99.6	98.8	99.6	99.6	99.6	98.8	99.8	98.8	99.8	98.8	98.8	99.8	99.8	99.8	99.8	99.7	99.8	97.5	99.8	99.8	100.0			
28	RVA/Human-wt/ZMB/UFS-NGS-MRC-DPRU16639/2016/G2P4	99.6	99.6	99.5	99.6	99.4	99.4	98.6	99.5	99.5	99.4	98.7	99.7	98.7	99.7	98.7	98.7	99.7	99.7	99.7	99.7	99.6	99.7	97.3	99.7	99.7	99.8	99.8		
29	RVA/Human-wt/ZMB/UFS-NGS-MRC-DPRU16646/2016/G2P4	99.8	99.8	99.7	99.8	99.7	99.7	98.8	99.8	99.7	99.7	99.0	99.9	99.0	99.9	99.0	99.0	99.9	99.9	99.9	99.9	99.8	99.9	97.5	99.9	99.9	99.8	99.8	99.7	

VP2

		1	2	3	4	5	6	7	8	9	10	11	12	13	14	15	16	17	18	19	20	21	22	23	24	25	26	27	28	29
1	RVA/Human-wt/ZMB/UFS-NGS-MRC-DPRU1724/2012/G2P4																													
2	RVA/Human-wt/ZMB/UFS-NGS-MRC-DPRU4718/2014/G2P4	99.8																												
3	RVA/Human-wt/ZMB/UFS-NGS-MRC-DPRU1729/2012/G2P4	100.0	99.9																											
4	RVA/Human-wt/ZMB/UFS-NGS-MRC-DPRU4691/2014/G2P4	99.8	100.0	99.9																										
5	RVA/Human-wt/ZMB/UFS-NGS-MRC-DPRU4699/2014/G2P4	100.0	99.9	100.0	99.9																									
6	RVA/Human-wt/ZMB/UFS-NGS-MRC-DPRU4705/2014/G2P4	99.8	100.0	99.9	100.0	99.9																								
7	RVA/Human-wt/ZMB/UFS-NGS-MRC-DPRU4714/2014/G2P4	99.8	100.0	99.9	100.0	99.9	100.0																							
8	RVA/Human-wt/ZMB/UFS-NGS-MRC-DPRU4736/2014/G2P4	100.0	99.9	100.0	99.9	100.0	99.9	99.9																						
9	RVA/Human-wt/ZMB/UFS-NGS-MRC-DPRU4770/2014/G2P4	99.9	99.8	100.0	99.8	100.0	99.8	99.8	100.0																					
10	RVA/Human-wt/ZMB/UFS-NGS-MRC-DPRU4819/2014/G2P4	99.8	100.0	99.9	100.0	99.9	100.0	100.0	99.9	99.8																				
11	RVA/Human-wt/ZMB/UFS-NGS-MRC-DPRU9393/2015/G2P4	99.8	99.7	99.8	99.7	99.8	99.7	99.7	99.8	99.8	99.7																			
12	RVA/Human-wt/ZMB/UFS-NGS-MRC-DPRU9420/2015/G2P4	99.7	99.6	99.7	99.6	99.7	99.6	99.6	99.7	99.7	99.6	99.9																		
13	RVA/Human-wt/ZMB/UFS-NGS-MRC-DPRU9438/2015/G2P4	99.7	99.7	99.8	99.7	99.8	99.7	99.7	99.8	99.7	99.7	100.0	99.9																	
14	RVA/Human-wt/ZMB/UFS-NGS-MRC-DPRU9464/2015/G2P4	99.8	99.7	99.8	99.7	99.8	99.7	99.7	99.8	99.8	99.7	99.9	99.8	99.9																
15	RVA/Human-wt/ZMB/UFS-NGS-MRC-DPRU9475/2015/G2P4	99.7	99.7	99.8	99.7	99.8	99.7	99.7	99.8	99.7	99.7	100.0	99.9	99.9	99.9															
16	RVA/Human-wt/ZMB/UFS-NGS-MRC-DPRU9495/2015/G2P4	99.8	99.7	99.8	99.7	99.8	99.7	99.7	99.8	99.8	99.7	100.0	99.9	100.0	99.9	100.0														
17	RVA/Human-wt/ZMB/UFS-NGS-MRC-DPRU9502/2015/G2P4	99.7	99.7	99.8	99.7	99.8	99.7	99.7	99.8	99.7	99.7	99.9	99.9	99.8	99.9	99.8	99.9													
18	RVA/Human-wt/ZMB/UFS-NGS-MRC-DPRU9503/2015/G2P4	99.8	99.7	99.8	99.7	99.8	99.7	99.7	99.8	99.8	99.7	99.9	99.8	99.9	99.9	99.9	99.9	100.0												
19	RVA/Human-wt/ZMB/UFS-NGS-MRC-DPRU9515/2015/G2P4	99.7	99.6	99.7	99.6	99.7	99.6	99.6	99.7	99.7	99.6	99.8	99.8	99.8	99.8	99.8	99.8	99.8	99.8											
20	RVA/Human-wt/ZMB/UFS-NGS-MRC-DPRU9556/2015/G2P4	99.7	99.6	99.7	99.6	99.7	99.6	99.6	99.7	99.7	99.6	99.8	99.8	99.8	99.8	99.8	99.8	99.9	99.9	99.8										
21	RVA/Human-wt/ZMB/UFS-NGS-MRC-DPRU9559/2015/G2P4	99.8	99.7	99.8	99.7	99.8	99.7	99.7	99.8	99.8	99.7	100.0	99.9	99.9	100.0	99.9	100.0	99.9	100.0	99.9	99.9									
22	RVA/Human-wt/ZMB/UFS-NGS-MRC-DPRU13231/2016/G2P4	99.7	99.6	99.7	99.6	99.7	99.6	99.6	99.7	99.7	99.6	99.8	99.8	99.8	99.8	99.8	99.8	99.9	99.9	99.8	99.8	99.9								
23	RVA/Human-wt/ZMB/UFS-NGS-MRC-DPRU13301/2016/G2P4	99.7	99.6	99.7	99.6	99.7	99.6	99.6	99.7	99.7	99.6	99.8	99.8	99.8	99.8	99.8	99.8	99.9	99.9	99.8	99.8	99.9	99.9							
24	RVA/Human-wt/ZMB/UFS-NGS-MRC-DPRU13352/2016/G2P4	99.6	99.5	99.7	99.5	99.7	99.5	99.5	99.7	99.6	99.5	99.8	99.7	99.7	99.8	99.7	99.8	99.9	99.8	99.7	99.8	99.8	99.8	99.8						
25	RVA/Human-wt/ZMB/UFS-NGS-MRC-DPRU13545/2016/G2P4	99.7	99.6	99.7	99.6	99.7	99.6	99.6	99.7	99.7	99.6	99.8	99.8	99.8	99.8	99.8	99.8	99.9	99.9	99.8	99.8	99.9	99.9	100.0	99.8					
26	RVA/Human-wt/ZMB/UFS-NGS-MRC-DPRU16599/2016/G2P4	99.6	99.5	99.7	99.5	99.7	99.5	99.5	99.7	99.6	99.5	99.7	99.7	99.7	99.7	99.7	99.7	99.7	99.7	99.7	99.7	99.8	99.7	99.7	99.6	99.7				
27	RVA/Human-wt/ZMB/UFS-NGS-MRC-DPRU16601/2016/G2P4	99.6	99.5	99.7	99.5	99.7	99.5	99.5	99.7	99.6	99.5	99.7	99.7	99.7	99.7	99.7	99.7	99.7	99.7	99.7	99.7	99.8	99.7	99.7	99.6	99.7	100.0			
28	RVA/Human-wt/ZMB/UFS-NGS-MRC-DPRU16639/2016/G2P4	99.6	99.5	99.6	99.5	99.6	99.5	99.5	99.6	99.6	99.5	99.7	99.6	99.7	99.7	99.7	99.7	99.7	99.7	99.6	99.6	99.7	99.6	99.6	99.5	99.6	100.0	100.0		
29	RVA/Human-wt/ZMB/UFS-NGS-MRC-DPRU16646/2016/G2P4	99.6	99.5	99.7	99.5	99.7	99.5	99.5	99.7	99.6	99.5	99.8	99.7	99.7	99.8	99.7	99.8	99.9	99.8	99.7	99.8	99.8	99.8	99.8	100.0	99.8	99.6	99.6	99.5	

VP3

		1	2	3	4	5	6	7	8	9	10	11	12	13	14	15	16	17	18	19	20	21	22	23	24	25	26	27	28
1	RVA/Human-wt/ZMB/UFS-NGS-MRC-DPRU1724/2012/G2P4																												
2	RVA/Human-wt/ZMB/UFS-NGS-MRC-DPRU4699/2014/G2P4	99.8																											
3	RVA/Human-wt/ZMB/UFS-NGS-MRC-DPRU4705/2014/G2P4	99.8	99.6																										
4	RVA/Human-wt/ZMB/UFS-NGS-MRC-DPRU4714/2014/G2P4	99.6	99.5	99.8																									
5	RVA/Human-wt/ZMB/UFS-NGS-MRC-DPRU4718/2014/G2P4	99.0	98.9	99.3	99.1																								
6	RVA/Human-wt/ZMB/UFS-NGS-MRC-DPRU4736/2014/G2P4	99.8	100.0	99.6	99.4	98.9																							
7	RVA/Human-wt/ZMB/UFS-NGS-MRC-DPRU4770/2014/G2P4	99.7	99.8	99.5	99.4	98.8	99.8																						
8	RVA/Human-wt/ZMB/UFS-NGS-MRC-DPRU4819/2014/G2P4	99.7	99.6	100.0	99.8	99.2	99.6	99.5																					
9	RVA/Human-wt/ZMB/UFS-NGS-MRC-DPRU9393/2015/G2P4	99.7	99.6	99.5	99.4	98.9	99.5	99.4	99.5																				
10	RVA/Human-wt/ZMB/UFS-NGS-MRC-DPRU9420/2015/G2P4	99.7	99.6	99.6	99.4	98.9	99.6	99.5	99.5	99.9																			
11	RVA/Human-wt/ZMB/UFS-NGS-MRC-DPRU9438/2015/G2P4	99.7	99.6	99.6	99.4	98.9	99.6	99.5	99.5	100.0	99.9																		
12	RVA/Human-wt/ZMB/UFS-NGS-MRC-DPRU9464/2015/G2P4	99.7	99.5	99.4	99.4	98.8	99.4	99.4	99.4	99.8	99.8	99.8																	
13	RVA/Human-wt/ZMB/UFS-NGS-MRC-DPRU9475/2015/G2P4	99.7	99.5	99.4	99.3	98.8	99.4	99.4	99.4	99.8	99.8	99.9	99.8																
14	RVA/Human-wt/ZMB/UFS-NGS-MRC-DPRU9495/2015/G2P4	99.8	99.6	99.6	99.4	98.9	99.6	99.5	99.5	99.9	99.9	99.9	99.9	99.9															
15	RVA/Human-wt/ZMB/UFS-NGS-MRC-DPRU9502/2015/G2P4	99.8	99.6	99.6	99.4	99.0	99.6	99.5	99.6	99.9	100.0	100.0	99.8	99.8	100.0														
16	RVA/Human-wt/ZMB/UFS-NGS-MRC-DPRU9503/2015/G2P4	99.8	99.6	99.6	99.4	99.0	99.6	99.5	99.6	99.9	100.0	100.0	99.8	99.8	100.0	100.0													
17	RVA/Human-wt/ZMB/UFS-NGS-MRC-DPRU1729/2012/G2P4	100.0	99.8	99.7	99.6	99.0	99.7	99.6	99.7	99.7	99.8	99.8	99.7	99.7	99.8	99.8	99.8												
18	RVA/Human-wt/ZMB/UFS-NGS-MRC-DPRU4691/2014/G2P4	99.8	99.6	99.9	99.8	99.2	99.5	99.4	99.9	99.4	99.5	99.5	99.4	99.4	99.6	99.5	99.5	99.7											
19	RVA/Human-wt/ZMB/UFS-NGS-MRC-DPRU9515/2015/G2P4	99.8	99.6	99.6	99.4	98.9	99.6	99.5	99.5	99.9	99.9	99.9	99.9	99.9	100.0	100.0	100.0	99.8	99.6										
20	RVA/Human-wt/ZMB/UFS-NGS-MRC-DPRU9556/2015/G2P4	99.8	99.6	99.6	99.4	99.0	99.6	99.5	99.6	99.9	100.0	100.0	99.8	99.8	100.0	100.0	100.0	99.8	99.5	100.0									
21	RVA/Human-wt/ZMB/UFS-NGS-MRC-DPRU9559/2015/G2P4	99.7	99.5	99.5	99.3	98.8	99.5	99.4	99.4	99.8	99.8	99.8	99.8	99.8	99.9	99.9	99.9	99.8	99.5	99.9	99.9								
22	RVA/Human-wt/ZMB/UFS-NGS-MRC-DPRU13231/2016/G2P4	99.7	99.5	99.5	99.3	98.8	99.5	99.4	99.4	99.8	99.8	99.8	99.8	99.8	99.9	99.9	99.9	99.8	99.5	99.9	99.9	99.8							
23	RVA/Human-wt/ZMB/UFS-NGS-MRC-DPRU13301/2016/G2P4	99.7	99.5	99.4	99.3	98.8	99.4	99.4	99.4	99.8	99.8	99.8	99.8	99.8	99.9	99.8	99.8	99.7	99.4	99.9	99.8	99.8	100.0						
24	RVA/Human-wt/ZMB/UFS-NGS-MRC-DPRU13352/2016/G2P4	99.6	99.5	99.4	99.3	98.8	99.4	99.4	99.4	99.8	99.8	99.8	99.7	99.7	99.8	99.8	99.8	99.6	99.4	99.8	99.8	99.7	99.7	99.7					
25	RVA/Human-wt/ZMB/UFS-NGS-MRC-DPRU13545/2016/G2P4	99.6	99.5	99.5	99.3	98.8	99.5	99.4	99.4	99.8	99.8	99.8	99.7	99.7	99.8	99.9	99.9	99.7	99.4	99.8	99.9	99.8	99.9	100.0	99.7				
26	RVA/Human-wt/ZMB/UFS-NGS-MRC-DPRU16599/2016/G2P4	99.7	99.6	99.5	99.4	98.9	99.5	99.4	99.5	99.8	99.9	99.9	99.8	99.8	99.9	99.9	99.9	99.7	99.4	99.9	99.9	99.8	99.8	99.8	99.8	99.8			
27	RVA/Human-wt/ZMB/UFS-NGS-MRC-DPRU16601/2016/G2P4	99.7	99.5	99.5	99.3	98.8	99.5	99.4	99.4	99.8	99.8	99.8	99.8	99.8	99.9	99.9	99.9	99.8	99.5	99.9	99.9	99.8	99.8	99.8	99.7	99.8	100.0		
28	RVA/Human-wt/ZMB/UFS-NGS-MRC-DPRU16639/2016/G2P4	99.7	99.6	99.5	99.4	98.9	99.5	99.4	99.5	99.8	99.9	99.9	99.8	99.8	99.9	99.9	99.9	99.7	99.4	99.9	99.9	99.8	99.8	99.8	99.8	99.8	100.0	100.0	
29	RVA/Human-wt/ZMB/UFS-NGS-MRC-DPRU16646/2016/G2P4	99.6	99.5	99.5	99.3	98.8	99.5	99.4	99.4	99.8	99.8	99.8	99.7	99.7	99.8	99.9	99.9	99.7	99.4	99.8	99.9	99.8	99.8	99.7	100.0	99.8	99.8	99.8	99.8

VP4

		1	2	3	4	5	6	7	8	9	10	11	12	13	14	15	16	17	18	19	20	21	22	23	24	25	26	27	28
1	RVA/Human-wt/ZMB/UFS-NGS-MRC-DPRU1724/2012/G2P4																												
2	RVA/Human-wt/ZMB/UFS-NGS-MRC-DPRU1729/2012/G2P4	100.0																											
3	RVA/Human-wt/ZMB/UFS-NGS-MRC-DPRU4691/2014/G2P4	99.8	99.8																										
4	RVA/Human-wt/ZMB/UFS-NGS-MRC-DPRU4699/2014/G2P4	99.9	99.9	99.7																									
5	RVA/Human-wt/ZMB/UFS-NGS-MRC-DPRU4714/2014/G2P4	99.8	99.8	99.9	99.7																								
6	RVA/Human-wt/ZMB/UFS-NGS-MRC-DPRU4718/2014/G2P4	99.8	99.8	99.9	99.7	99.9																							
7	RVA/Human-wt/ZMB/UFS-NGS-MRC-DPRU4736/2014/G2P4	99.9	99.9	99.7	100.0	99.7	99.7																						
8	RVA/Human-wt/ZMB/UFS-NGS-MRC-DPRU4705/2014/G2P4	99.9	99.9	99.9	99.8	100.0	100.0	99.7																					
9	RVA/Human-wt/ZMB/UFS-NGS-MRC-DPRU4770/2014/G2P4	99.7	99.7	99.5	99.8	99.6	99.6	99.8	99.6																				
10	RVA/Human-wt/ZMB/UFS-NGS-MRC-DPRU4819/2014/G2P4	99.8	99.8	99.9	99.7	99.9	99.9	99.7	100.0	99.6																			
11	RVA/Human-wt/ZMB/UFS-NGS-MRC-DPRU9393/2015/G2P4	99.9	99.9	99.7	99.8	99.7	99.7	99.7	99.7	99.6	99.7																		
12	RVA/Human-wt/ZMB/UFS-NGS-MRC-DPRU9420/2015/G2P4	99.9	99.9	99.7	99.8	99.7	99.7	99.7	99.7	99.6	99.7	99.8																	
13	RVA/Human-wt/ZMB/UFS-NGS-MRC-DPRU9438/2015/G2P4	99.8	99.8	99.6	99.7	99.7	99.7	99.7	99.7	99.6	99.7	100.0	99.8																
14	RVA/Human-wt/ZMB/UFS-NGS-MRC-DPRU9464/2015/G2P4	99.9	99.9	99.7	99.8	99.7	99.7	99.7	99.7	99.6	99.7	99.8	99.8	99.8															
15	RVA/Human-wt/ZMB/UFS-NGS-MRC-DPRU9475/2015/G2P4	99.8	99.8	99.6	99.7	99.7	99.7	99.7	99.7	99.6	99.7	100.0	99.8	99.9	99.8														
16	RVA/Human-wt/ZMB/UFS-NGS-MRC-DPRU9502/2015/G2P4	99.9	99.9	99.7	99.8	99.7	99.7	99.8	99.8	99.7	99.7	99.9	99.9	99.8	99.9	99.8													
17	RVA/Human-wt/ZMB/UFS-NGS-MRC-DPRU9503/2015/G2P4	99.9	99.9	99.7	99.8	99.7	99.7	99.7	99.7	99.6	99.7	99.8	99.8	99.8	99.8	99.8	99.9												
18	RVA/Human-wt/ZMB/UFS-NGS-MRC-DPRU9495/2015/G2P4	99.9	99.9	99.7	99.8	99.7	99.7	99.7	99.7	99.6	99.7	100.0	99.8	100.0	99.8	100.0	99.9	99.8											
19	RVA/Human-wt/ZMB/UFS-NGS-MRC-DPRU9515/2015/G2P4	100.0	100.0	99.7	99.9	99.8	99.8	99.8	99.8	99.7	99.8	99.9	99.9	99.9	99.9	99.9	100.0	99.9	99.9										
20	RVA/Human-wt/ZMB/UFS-NGS-MRC-DPRU9556/2015/G2P4	100.0	100.0	99.7	99.9	99.8	99.8	99.8	99.8	99.7	99.8	99.9	99.9	99.9	99.9	99.9	100.0	99.9	99.9										
21	RVA/Human-wt/ZMB/UFS-NGS-MRC-DPRU9559/2015/G2P4	99.9	99.9	99.7	99.8	99.7	99.7	99.7	99.7	99.6	99.7	99.8	99.8	99.8	99.8	99.8	99.9	99.8	99.8	99.9	99.9								
22	RVA/Human-wt/ZMB/UFS-NGS-MRC-DPRU13231/2016/G2P4	99.7	99.7	99.4	99.6	99.5	99.5	99.6	99.5	99.4	99.5	99.6	99.6	99.6	99.6	99.6	99.7	99.6	99.6	99.7	99.7	99.6							
23	RVA/Human-wt/ZMB/UFS-NGS-MRC-DPRU13301/2016/G2P4	99.7	99.7	99.5	99.6	99.5	99.5	99.7	99.6	99.4	99.5	99.7	99.7	99.6	99.7	99.6	99.7	99.7	99.7	99.7	99.7	99.7	99.9						
24	RVA/Human-wt/ZMB/UFS-NGS-MRC-DPRU13352/2016/G2P4	99.8	99.8	99.6	99.7	99.7	99.7	99.7	99.7	99.6	99.7	99.8	99.8	99.7	99.8	99.7	99.8	99.8	99.8	99.9	99.9	99.8	99.6	99.6					
25	RVA/Human-wt/ZMB/UFS-NGS-MRC-DPRU13545/2016/G2P4	99.7	99.7	99.4	99.6	99.5	99.5	99.6	99.5	99.4	99.5	99.6	99.6	99.6	99.6	99.6	99.7	99.6	99.6	99.7	99.7	99.6	99.8	100.0	99.6				
26	RVA/Human-wt/ZMB/UFS-NGS-MRC-DPRU16599/2016/G2P4	99.7	99.7	99.5	99.7	99.6	99.6	99.6	99.6	99.5	99.6	99.7	99.7	99.7	99.7	99.7	99.7	99.7	99.7	99.8	99.8	99.7	99.5	99.5	99.7	99.5			
27	RVA/Human-wt/ZMB/UFS-NGS-MRC-DPRU16639/2016/G2P4	99.8	99.8	99.6	99.7	99.7	99.7	99.7	99.7	99.6	99.7	99.8	99.8	99.7	99.8	99.7	99.8	99.8	99.8	99.9	99.9	99.8	99.6	99.6	99.7	99.6	99.8		
28	RVA/Human-wt/ZMB/UFS-NGS-MRC-DPRU16601/2016/G2P4	99.7	99.7	99.5	99.7	99.6	99.6	99.6	99.6	99.5	99.6	99.7	99.7	99.7	99.7	99.7	99.7	99.7	99.7	99.8	99.8	99.7	99.5	99.5	99.7	99.5	100.0	99.8	
29	RVA/Human-wt/ZMB/UFS-NGS-MRC-DPRU16646/2016/G2P4	99.8	99.8	99.6	99.7	99.7	99.7	99.7	99.7	99.6	99.7	99.8	99.8	99.7	99.8	99.7	99.8	99.8	99.8	99.9	99.9	99.8	99.6	99.6	100.0	99.6	99.7	99.7	99.7

VP6

		1	2	3	4	5	6	7	8	9	10	11	12	13	14	15	16	17	18	19	20	21	22	23	24	25	26	27	28	29
1	RVA/Human-wt/ZMB/UFS-NGS-MRC-DPRU1724/2012/G2P4																													
2	RVA/Human-wt/ZMB/UFS-NGS-MRC-DPRU4699/2014/G2P4	97.6																												
3	RVA/Human-wt/ZMB/UFS-NGS-MRC-DPRU4705/2014/G2P4	99.9	97.6																											
4	RVA/Human-wt/ZMB/UFS-NGS-MRC-DPRU4714/2014/G2P4	99.7	97.6	99.8																										
5	RVA/Human-wt/ZMB/UFS-NGS-MRC-DPRU4718/2014/G2P4	99.9	97.6	100.0	99.8																									
6	RVA/Human-wt/ZMB/UFS-NGS-MRC-DPRU4736/2014/G2P4	97.6	100.0	97.6	97.6	97.6																								
7	RVA/Human-wt/ZMB/UFS-NGS-MRC-DPRU4770/2014/G2P4	97.6	99.9	97.6	97.6	97.6	99.9																							
8	RVA/Human-wt/ZMB/UFS-NGS-MRC-DPRU4819/2014/G2P4	99.9	97.6	100.0	99.8	100.0	97.6	97.6																						
9	RVA/Human-wt/ZMB/UFS-NGS-MRC-DPRU9393/2015/G2P4	99.7	97.4	99.7	99.5	99.7	97.4	97.4	99.7																					
10	RVA/Human-wt/ZMB/UFS-NGS-MRC-DPRU9420/2015/G2P4	99.8	97.5	99.7	99.6	99.7	97.5	97.5	99.7	99.9																				
11	RVA/Human-wt/ZMB/UFS-NGS-MRC-DPRU9438/2015/G2P4	99.7	97.4	99.7	99.5	99.7	97.4	97.4	99.7	100.0	99.9																			
12	RVA/Human-wt/ZMB/UFS-NGS-MRC-DPRU9464/2015/G2P4	99.8	97.5	99.7	99.6	99.7	97.5	97.5	99.7	99.9	100.0	99.9																		
13	RVA/Human-wt/ZMB/UFS-NGS-MRC-DPRU9475/2015/G2P4	99.7	97.4	99.7	99.5	99.7	97.4	97.4	99.7	100.0	99.9	100.0	99.9																	
14	RVA/Human-wt/ZMB/UFS-NGS-MRC-DPRU9495/2015/G2P4	99.7	97.4	99.7	99.5	99.7	97.4	97.4	99.7	100.0	99.9	100.0	99.9	100.0																
15	RVA/Human-wt/ZMB/UFS-NGS-MRC-DPRU9502/2015/G2P4	99.8	97.5	99.7	99.6	99.7	97.5	97.5	99.7	99.9	100.0	99.9	100.0	99.9	99.9															
16	RVA/Human-wt/ZMB/UFS-NGS-MRC-DPRU9503/2015/G2P4	99.8	97.5	99.7	99.6	99.7	97.5	97.5	99.7	99.9	100.0	99.9	100.0	99.9	99.9	100.0														
17	RVA/Human-wt/ZMB/UFS-NGS-MRC-DPRU9515/2015/G2P4	99.8	97.5	99.7	99.6	99.7	97.5	97.5	99.7	99.9	100.0	99.9	100.0	99.9	99.9	100.0	100.0													
18	RVA/Human-wt/ZMB/UFS-NGS-MRC-DPRU9556/2015/G2P4	99.8	97.5	99.7	99.6	99.7	97.5	97.5	99.7	99.9	100.0	99.9	100.0	99.9	99.9	100.0	100.0	100.0												
19	RVA/Human-wt/ZMB/UFS-NGS-MRC-DPRU9559/2015/G2P4	99.8	97.5	99.7	99.6	99.7	97.5	97.5	99.7	99.9	100.0	99.9	100.0	99.9	99.9	100.0	100.0	100.0	100.0											
20	RVA/Human-wt/ZMB/UFS-NGS-MRC-DPRU13231/2016/G2P4	99.8	97.5	99.7	99.6	99.7	97.5	97.5	99.7	99.9	100.0	99.9	100.0	99.9	99.9	100.0	100.0	100.0	100.0	100.0										
21	RVA/Human-wt/ZMB/UFS-NGS-MRC-DPRU13301/2016/G2P4	99.8	97.5	99.7	99.6	99.7	97.5	97.5	99.7	99.9	100.0	99.9	100.0	99.9	99.9	100.0	100.0	100.0	100.0	100.0	100.0									
22	RVA/Human-wt/ZMB/UFS-NGS-MRC-DPRU13352/2016/G2P4	99.8	97.5	99.7	99.6	99.7	97.5	97.5	99.7	99.9	100.0	99.9	100.0	99.9	99.9	100.0	100.0	100.0	100.0	100.0	100.0	100.0								
23	RVA/Human-wt/ZMB/UFS-NGS-MRC-DPRU13545/2016/G2P4	99.8	97.5	99.7	99.6	99.7	97.5	97.5	99.7	99.9	100.0	99.9	100.0	99.9	99.9	100.0	100.0	100.0	100.0	100.0	100.0	100.0	100.0							
24	RVA/Human-wt/ZMB/UFS-NGS-MRC-DPRU16599/2016/G2P4	99.3	97.0	99.2	99.1	99.2	97.0	97.0	99.2	99.4	99.5	99.4	99.5	99.4	99.4	99.5	99.5	99.5	99.5	99.5	99.5	99.5	99.5	99.5						
25	RVA/Human-wt/ZMB/UFS-NGS-MRC-DPRU16601/2016/G2P4	99.7	97.3	99.6	99.4	99.6	97.3	97.3	99.6	99.7	99.8	99.7	99.8	99.7	99.7	99.8	99.8	99.8	99.8	99.8	99.8	99.8	99.8	99.8	99.7					
26	RVA/Human-wt/ZMB/UFS-NGS-MRC-DPRU16699/2016/G2P4	99.6	97.2	99.5	99.3	99.5	97.2	97.2	99.5	99.7	99.7	99.7	99.7	99.7	99.7	99.7	99.7	99.7	99.7	99.7	99.7	99.7	99.7	99.7	99.6	99.9				
27	RVA/Human-wt/ZAM/UFS-NGS-MRC-DPRU1729/2012/G2P4	99.8	97.5	99.7	99.6	99.7	97.5	97.5	99.7	99.6	99.7	99.6	99.7	99.6	99.6	99.7	99.7	99.7	99.7	99.7	99.7	99.7	99.7	99.7	99.3	99.5	99.4			
28	RVA/Human-wt/ZMB/UFS-NGS-MRC-DPRU4699/2014/G2P4	99.9	97.6	100.0	99.8	100.0	97.6	97.6	100.0	99.7	99.7	99.7	99.7	99.7	99.7	99.7	99.7	99.7	99.7	99.7	99.7	99.7	99.7	99.7	99.2	99.6	99.5	99.7		
29	RVA/Human-wt/ZMB/UFS-NGS-MRC-DPRU16646/2016/G2P4	99.7	97.3	99.6	99.4	99.6	97.3	97.3	99.6	99.7	99.8	99.7	99.8	99.7	99.7	99.8	99.8	99.8	99.8	99.8	99.8	99.8	99.8	99.8	99.6	99.7	99.6	99.5	99.6	

VP7

		1	2	3	4	5	6	7	8	9	10	11	12	13	14	15	16	17	18	19	20	21	22	23	24	25	26	27	28	29
1	RVA/Human-wt/ZAM/UFS-NGS-MRC-DPRU1724/2012/G2P4																													
2	RVA/Human-wt/ZAM/UFS-NGS-MRC-DPRU1729/2012/G2P4	100.0																												
3	RVA/Human-wt/ZAM/UFS-NGS-MRC-DPRU4691/2014/G2P4	100.0	100.0																											
4	RVA/Human-wt/ZAM/UFS-NGS-MRC-DPRU4699/2014/G2P4	99.9	99.9	99.9																										
5	RVA/Human-wt/ZAM/UFS-NGS-MRC-DPRU4705/2014/G2P4	100.0	100.0	100.0	99.9																									
6	RVA/Human-wt/ZAM/UFS-NGS-MRC-DPRU4714/2014/G2P4	100.0	100.0	100.0	99.9	100.0																								
7	RVA/Human-wt/ZAM/UFS-NGS-MRC-DPRU4718/2014/G2P4	97.6	97.6	97.6	97.5	97.6	97.6																							
8	RVA/Human-wt/ZAM/UFS-NGS-MRC-DPRU4736/2014/G2P4	99.9	99.9	99.9	100.0	99.9	99.9	97.5																						
9	RVA/Human-wt/ZAM/UFS-NGS-MRC-DPRU4770/2014/G2P4	100.0	100.0	100.0	99.9	100.0	100.0	97.6	99.9																					
10	RVA/Human-wt/ZAM/UFS-NGS-MRC-DPRU4819/2014/G2P4	100.0	100.0	100.0	99.9	100.0	100.0	97.6	99.9	100.0																				
11	RVA/Human-wt/ZAM/UFS-NGS-MRC-DPRU9393/2015/G2P4	99.9	99.9	99.9	99.8	99.9	99.9	97.5	99.8	99.9	99.9																			
12	RVA/Human-wt/ZAM/UFS-NGS-MRC-DPRU9420/2015/G2P4	99.9	99.9	99.9	99.8	99.9	99.9	97.5	99.8	99.9	99.9	100.0																		
13	RVA/Human-wt/ZAM/UFS-NGS-MRC-DPRU9438/2015/G2P4	99.9	99.9	99.9	99.8	99.9	99.9	97.5	99.8	99.9	99.9	100.0	100.0																	
14	RVA/Human-wt/ZAM/UFS-NGS-MRC-DPRU9464/2015/G2P4	99.7	99.7	99.7	99.6	99.7	99.7	97.3	99.6	99.7	99.7	99.8	99.8	99.8																
15	RVA/Human-wt/ZAM/UFS-NGS-MRC-DPRU9475/2015/G2P4	99.9	99.9	99.9	99.8	99.9	99.9	97.5	99.8	99.9	99.9	100.0	100.0	100.0	99.8															
16	RVA/Human-wt/ZAM/UFS-NGS-MRC-DPRU9495/2015/G2P4	99.9	99.9	99.9	99.8	99.9	99.9	97.5	99.8	99.9	99.9	100.0	100.0	100.0	99.8	100.0														
17	RVA/Human-wt/ZAM/UFS-NGS-MRC-DPRU9502/2015/G2P4	99.9	99.9	99.9	99.8	99.9	99.9	97.5	99.8	99.9	99.9	100.0	100.0	100.0	99.8	100.0	100.0													
18	RVA/Human-wt/ZAM/UFS-NGS-MRC-DPRU9503/2015/G2P4	99.8	99.8	99.8	99.7	99.8	99.8	97.4	99.7	99.8	99.8	99.9	99.9	99.9	99.7	99.9	99.9	99.9												
19	RVA/Human-wt/ZAM/UFS-NGS-MRC-DPRU9515/2015/G2P4	99.9	99.9	99.9	99.8	99.9	99.9	97.5	99.8	99.9	99.9	100.0	100.0	100.0	99.8	100.0	100.0	100.0	99.9											
20	RVA/Human-wt/ZAM/UFS-NGS-MRC-DPRU9556/2015/G2P4	99.9	99.9	99.9	99.8	99.9	99.9	97.5	99.8	99.9	99.9	100.0	100.0	100.0	99.8	100.0	100.0	100.0	99.9	100.0										
21	RVA/Human-wt/ZAM/UFS-NGS-MRC-DPRU9559/2015/G2P4	99.8	99.8	99.8	99.7	99.8	99.8	97.4	99.7	99.8	99.8	99.9	99.9	99.9	99.7	99.9	99.9	99.9	99.8	99.9	99.9									
22	RVA/Human-wt/ZAM/UFS-NGS-MRC-DPRU13231/2016/G2P4	99.7	99.7	99.7	99.6	99.7	99.7	97.3	99.6	99.7	99.7	99.8	99.8	99.8	99.6	99.8	99.8	99.8	99.7	99.8	99.8	99.7								
23	RVA/Human-wt/ZAM/UFS-NGS-MRC-DPRU13301/2016/G2P4	99.6	99.6	99.6	99.5	99.6	99.6	97.2	99.5	99.6	99.6	99.7	99.7	99.7	99.5	99.7	99.7	99.7	99.6	99.7	99.7	99.6	99.7							
24	RVA/Human-wt/ZAM/UFS-NGS-MRC-DPRU13352/2016/G2P4	99.8	99.8	99.8	99.7	99.8	99.8	97.6	99.7	99.8	99.8	99.9	99.9	99.9	99.7	99.9	99.9	99.9	99.8	99.9	99.9	99.8	99.7	99.6						
25	RVA/Human-wt/ZAM/UFS-NGS-MRC-DPRU13545/2016/G2P4	99.5	99.5	99.5	99.4	99.5	99.5	97.1	99.4	99.5	99.5	99.6	99.6	99.6	99.4	99.6	99.6	99.6	99.5	99.6	99.6	99.5	99.6	99.9	99.5					
26	RVA/Human-wt/ZAM/UFS-NGS-MRC-DPRU16599/2016/G2P4	99.7	99.7	99.7	99.6	99.7	99.7	97.3	99.6	99.7	99.7	99.8	99.8	99.8	99.6	99.8	99.8	99.8	99.7	99.8	99.8	99.7	99.6	99.5	99.7	99.4				
27	RVA/Human-wt/ZAM/UFS-NGS-MRC-DPRU16601/2016/G2P4	99.7	99.7	99.7	99.6	99.7	99.7	97.3	99.6	99.7	99.7	99.8	99.8	99.8	99.6	99.8	99.8	99.8	99.7	99.8	99.8	99.7	99.6	99.5	99.7	99.4	100.0			
28	RVA/Human-wt/ZAM/UFS-NGS-MRC-DPRU16639/2016/G2P4	99.7	99.7	99.7	99.6	99.7	99.7	97.3	99.6	99.7	99.7	99.8	99.8	99.8	99.6	99.8	99.8	99.8	99.7	99.8	99.8	99.7	99.6	99.5	99.7	99.4	99.8	99.8		
29	RVA/Human-wt/ZAM/UFS-NGS-MRC-DPRU16646/2016/G2P4	99.8	99.8	99.8	99.7	99.8	99.8	97.6	99.7	99.8	99.8	99.9	99.9	99.9	99.7	99.9	99.9	99.9	99.8	99.9	99.9	99.8	99.7	99.6	100.0	99.5	99.7	99.7	99.7	

NSP1

1	RVA/Human-wt/ZMB/UFS-NGS-MRC-DPRU13231/2016/G2P4																													
2	RVA/Human-wt/ZMB/UFS-NGS-MRC-DPRU13301/2016/G2P4	99.9																												
3	RVA/Human-wt/ZMB/UFS-NGS-MRC-DPRU13352/2016/G2P4	99.7	99.8																											
4	RVA/Human-wt/ZMB/UFS-NGS-MRC-DPRU13545/2016/G2P4	99.9	99.9	99.7																										
5	RVA/Human-wt/ZMB/UFS-NGS-MRC-DPRU16599/2016/G2P4	99.6	99.7	99.6	99.6																									
6	RVA/Human-wt/ZMB/UFS-NGS-MRC-DPRU16601/2016/G2P4	99.6	99.7	99.6	99.6	100.0																								
7	RVA/Human-wt/ZMB/UFS-NGS-MRC-DPRU16639/2016/G2P4	99.6	99.7	99.6	99.6	99.9	99.9																							
8	RVA/Human-wt/ZMB/UFS-NGS-MRC-DPRU16646/2016/G2P4	99.7	99.8	100.0	99.7	99.6	99.6	99.6																						
9	RVA/Human-wt/ZMB/UFS-NGS-MRC-DPRU1724/2012/G2P4	99.7	99.7	99.7	99.7	99.5	99.5	99.5	99.7																					
10	RVA/Human-wt/ZMB/UFS-NGS-MRC-DPRU1729/2012/G2P4	99.6	99.7	99.6	99.6	99.5	99.5	99.5	99.6	99.8																				
11	RVA/Human-wt/ZMB/UFS-NGS-MRC-DPRU4691/2014/G2P4	99.5	99.5	99.6	99.5	99.3	99.3	99.3	99.6	99.7	99.6																			
12	RVA/Human-wt/ZMB/UFS-NGS-MRC-DPRU4699/2014/G2P4	99.5	99.6	99.5	99.5	99.4	99.4	99.4	99.5	99.9	99.7	99.5																		
13	RVA/Human-wt/ZMB/UFS-NGS-MRC-DPRU4705/2014/G2P4	99.5	99.5	99.6	99.5	99.3	99.3	99.3	99.6	99.7	99.6	100.0	99.5																	
14	RVA/Human-wt/ZMB/UFS-NGS-MRC-DPRU4714/2014/G2P4	99.5	99.5	99.6	99.5	99.3	99.3	99.3	99.6	99.7	99.6	100.0	99.5	100.0																
15	RVA/Human-wt/ZMB/UFS-NGS-MRC-DPRU4718/2014/G2P4	99.3	99.4	99.5	99.3	99.2	99.2	99.2	99.5	99.5	99.5	99.9	99.4	99.9	99.9															
16	RVA/Human-wt/ZMB/UFS-NGS-MRC-DPRU4736/2014/G2P4	99.5	99.6	99.5	99.5	99.4	99.4	99.4	99.5	99.9	99.7	99.5	100.0	99.5	99.5	99.4														
17	RVA/Human-wt/ZMB/UFS-NGS-MRC-DPRU4770/2014/G2P4	99.6	99.7	99.6	99.6	99.5	99.5	99.5	99.6	99.9	99.7	99.6	99.9	99.6	99.6	99.5	99.9													
18	RVA/Human-wt/ZMB/UFS-NGS-MRC-DPRU9495/2015/G2P4	99.8	99.9	99.8	99.8	99.7	99.7	99.7	99.8	99.9	99.8	99.7	99.7	99.7	99.7	99.5	99.7	99.8												
19	RVA/Human-wt/ZMB/UFS-NGS-MRC-DPRU9438/2015/G2P4	99.8	99.9	99.8	99.8	99.7	99.7	99.7	99.8	99.9	99.8	99.7	99.7	99.7	99.7	99.5	99.7	99.8	100.0											
20	RVA/Human-wt/ZMB/UFS-NGS-MRC-DPRU9393/2015/G2P4	99.8	99.9	99.8	99.8	99.7	99.7	99.7	99.8	99.9	99.8	99.7	99.7	99.7	99.7	99.5	99.7	99.8	100.0	100.0										
21	RVA/Human-wt/ZMB/UFS-NGS-MRC-DPRU9407/2015/G2P4	99.8	99.9	99.8	99.8	99.7	99.7	99.7	99.8	99.9	99.8	99.7	99.7	99.7	99.7	99.5	99.7	99.8	100.0	100.0	100.0									
22	RVA/Human-wt/ZMB/UFS-NGS-MRC-DPRU9420/2015/G2P4	99.7	99.7	99.7	99.7	99.5	99.5	99.5	99.7	99.6	99.5	99.4	99.5	99.4	99.4	99.3	99.5	99.5	99.7	99.7	99.7	99.7								
23	RVA/Human-wt/ZMB/UFS-NGS-MRC-DPRU9464/2015/G2P4	99.8	99.9	99.8	99.8	99.7	99.7	99.7	99.8	99.7	99.7	99.5	99.6	99.5	99.5	99.4	99.6	99.7	99.9	99.9	99.9	99.9	99.7							
24	RVA/Human-wt/ZMB/UFS-NGS-MRC-DPRU9475/2015/G2P4	99.7	99.8	99.7	99.7	99.6	99.6	99.6	99.7	99.8	99.7	99.6	99.7	99.6	99.6	99.5	99.7	99.7	99.9	99.9	99.9	99.9	99.7	99.8						
25	RVA/Human-wt/ZMB/UFS-NGS-MRC-DPRU9502/2015/G2P4	99.8	99.9	99.8	99.8	99.7	99.7	99.7	99.8	99.7	99.7	99.5	99.6	99.5	99.5	99.4	99.6	99.7	99.9	99.9	99.9	99.9	99.7	99.9	99.8					
26	RVA/Human-wt/ZMB/UFS-NGS-MRC-DPRU9503/2014/G2P4	99.9	99.9	99.9	99.9	99.7	99.7	99.7	99.9	99.8	99.7	99.6	99.7	99.6	99.6	99.5	99.7	99.7	99.9	99.9	99.9	99.9	99.8	99.9	99.9	99.9				
27	RVA/Human-wt/ZMB/UFS-NGS-MRC-DPRU9515/2015/G2P4	99.9	99.9	99.9	99.9	99.7	99.7	99.7	99.9	99.8	99.7	99.6	99.7	99.6	99.6	99.5	99.7	99.7	99.9	99.9	99.9	99.9	99.8	99.9	99.9	99.9	100.0			
28	RVA/Human-wt/ZMB/UFS-NGS-MRC-DPRU9556/2015/G2P4	99.8	99.9	99.8	99.8	99.7	99.7	99.7	99.8	99.7	99.7	99.5	99.6	99.5	99.5	99.4	99.6	99.7	99.9	99.9	99.9	99.9	99.7	99.9	99.8	99.9	99.9	99.9		
29	RVA/Human-wt/ZMB/UFS-NGS-MRC-DPRU9559/2015/G2P4	99.7	99.8	99.7	99.7	99.6	99.6	99.6	99.7	99.8	99.7	99.6	99.7	99.6	99.6	99.5	99.7	99.7	99.9	99.9	99.9	99.9	99.7	99.8	99.9	99.8	99.9	99.9	99.8	

NSP2

		1	2	3	4	5	6	7	8	9	10	11	12	13	14	15	16	17	18	19	20	21	22	23	24	25	26	27	28	29
1	RVA/Human-wt/ZMB/UFS-NGS-MRC-DPRU1724/2012/G2P4																													
2	RVA/Human-wt/ZMB/UFS-NGS-MRC-DPRU1729/2012/G2P4	100.0																												
3	RVA/Human-wt/ZMB/UFS-NGS-MRC-DPRU4691/2014/G2P4	99.8	99.8																											
4	RVA/Human-wt/ZMB/UFS-NGS-MRC-DPRU4699/2014/G2P4	99.9	99.9	99.7																										
5	RVA/Human-wt/ZMB/UFS-NGS-MRC-DPRU4705/2014/G2P4	99.9	99.9	99.9	99.8																									
6	RVA/Human-wt/ZMB/UFS-NGS-MRC-DPRU4714/2014/G2P4	99.9	99.9	99.9	99.8	100.0																								
7	RVA/Human-wt/ZMB/UFS-NGS-MRC-DPRU4718/2014/G2P4	99.8	99.8	99.8	99.7	99.9	99.9																							
8	RVA/Human-wt/ZMB/UFS-NGS-MRC-DPRU4736/2014/G2P4	99.9	99.9	99.7	100.0	99.8	99.8	99.7																						
9	RVA/Human-wt/ZMB/UFS-NGS-MRC-DPRU4770/2012/G2P4	99.8	99.8	99.6	99.9	99.7	99.7	99.6	99.9																					
10	RVA/Human-wt/ZMB/UFS-NGS-MRC-DPRU4819/2014/G2P4	99.8	99.8	99.8	99.7	99.9	99.9	99.8	99.7	99.6																				
11	RVA/Human-wt/ZMB/UFS-NGS-MRC-DPRU9393/2015/G2P4	100.0	100.0	99.8	99.9	99.9	99.9	99.8	99.9	99.8	99.8																			
12	RVA/Human-wt/ZMB/UFS-NGS-MRC-DPRU9420/2015/G2P4	100.0	100.0	99.8	99.9	99.9	99.9	99.8	99.9	99.8	99.8	100.0																		
13	RVA/Human-wt/ZMB/UFS-NGS-MRC-DPRU9438/2015/G2P4	100.0	100.0	99.8	99.9	99.9	99.9	99.8	99.9	99.8	99.8	100.0	100.0																	
14	RVA/Human-wt/ZMB/UFS-NGS-MRC-DPRU9464/2015/G2P4	100.0	100.0	99.8	99.9	99.9	99.9	99.8	99.9	99.8	99.8	100.0	100.0	100.0																
15	RVA/Human-wt/ZMB/UFS-NGS-MRC-DPRU9475/2015/G2P4	100.0	100.0	99.8	99.9	99.9	99.9	99.8	99.9	99.8	99.8	100.0	100.0	100.0	100.0															
16	RVA/Human-wt/ZMB/UFS-NGS-MRC-DPRU9495/2015/G2P4	100.0	100.0	99.8	99.9	99.9	99.9	99.8	99.9	99.8	99.8	100.0	100.0	100.0	100.0	100.0														
17	RVA/Human-wt/ZMB/UFS-NGS-MRC-DPRU9503/2015/G2P4	100.0	100.0	99.8	99.9	99.9	99.9	99.8	99.9	99.8	99.8	100.0	100.0	100.0	100.0	100.0	100.0													
18	RVA/Human-wt/ZMB/UFS-NGS-MRC-DPRU9502/2015/G2P4	100.0	100.0	99.8	99.9	99.9	99.9	99.8	99.9	99.8	99.8	100.0	100.0	100.0	100.0	100.0	100.0	100.0												
19	RVA/Human-wt/ZMB/UFS-NGS-MRC-DPRU9515/2015/G2P4	100.0	100.0	99.8	99.9	99.9	99.9	99.8	99.9	99.8	99.8	100.0	100.0	100.0	100.0	100.0	100.0	100.0	100.0											
20	RVA/Human-wt/ZMB/UFS-NGS-MRC-DPRU9556/2015/G2P4	99.9	99.9	99.7	99.8	99.8	99.8	99.7	99.8	99.7	99.7	99.9	99.9	99.9	99.9	99.9	99.9	99.9	99.9	99.9										
21	RVA/Human-wt/ZMB/UFS-NGS-MRC-DPRU9559/2015/G2P4	100.0	100.0	99.8	99.9	99.9	99.9	99.8	99.9	99.8	99.8	100.0	100.0	100.0	100.0	100.0	100.0	100.0	100.0	100.0	99.9									
22	RVA/Human-wt/ZMB/UFS-NGS-MRC-DPRU13231/2016/G2P4	99.8	99.8	99.6	99.7	99.7	99.7	99.6	99.7	99.6	99.6	99.8	99.8	99.8	99.8	99.8	99.8	99.8	99.8	99.8	99.7	99.8								
23	RVA/Human-wt/ZMB/UFS-NGS-MRC-DPRU13301/2016/G2P4	99.8	99.8	99.6	99.7	99.7	99.7	99.6	99.7	99.6	99.6	99.8	99.8	99.8	99.8	99.8	99.8	99.8	99.8	99.8	99.7	99.8	100.0							
24	RVA/Human-wt/ZMB/UFS-NGS-MRC-DPRU13352/2016/G2P4	99.9	99.9	99.7	99.8	99.8	99.8	99.7	99.8	99.7	99.7	99.9	99.9	99.9	99.9	99.9	99.9	99.9	99.9	99.9	99.8	99.9	99.7	99.7						
25	RVA/Human-wt/ZMB/UFS-NGS-MRC-DPRU13545/2016/G2P4	99.8	99.8	99.6	99.7	99.7	99.7	99.6	99.7	99.6	99.6	99.8	99.8	99.8	99.8	99.8	99.8	99.8	99.8	99.8	99.7	99.8	100.0	100.0	99.7					
26	RVA/Human-wt/ZMB/UFS-NGS-MRC-DPRU16599/2016/G2P4	99.9	99.9	99.7	99.8	99.8	99.8	99.7	99.8	99.7	99.7	99.9	99.9	99.9	99.9	99.9	99.9	99.9	99.9	99.9	99.8	99.9	99.7	99.7	99.8	99.7				
27	RVA/Human-wt/ZMB/UFS-NGS-MRC-DPRU16601/2016/G2P4	99.9	99.9	99.7	99.8	99.8	99.8	99.7	99.8	99.7	99.7	99.9	99.9	99.9	99.9	99.9	99.9	99.9	99.9	99.9	99.8	99.9	99.7	99.7	99.8	99.7	100.0			
28	RVA/Human-wt/ZMB/UFS-NGS-MRC-DPRU16639/2016/G2P4	99.8	99.8	99.6	99.7	99.7	99.7	99.6	99.7	99.6	99.6	99.8	99.8	99.8	99.8	99.8	99.8	99.8	99.8	99.8	99.7	99.8	99.6	99.6	99.7	99.6	99.7	99.7		
29	RVA/Human-wt/ZMB/UFS-NGS-MRC-DPRU16646/2016/G2P4	99.9	99.9	99.7	99.8	99.8	99.8	99.7	99.8	99.7	99.7	99.9	99.9	99.9	99.9	99.9	99.9	99.9	99.9	99.9	99.8	99.9	99.7	99.7	100.0	99.7	99.8	99.8	99.7	

NSP3

		1	2	3	4	5	6	7	8	9	10	11	12	13	14	15	16	17	18	19	20	21	22	23	24	25	26	27	28	29
1	RVA/Human-wt/ZMB/UFS-NGS-MRC-DPRU1724/2012/G2P4																													
2	RVA/Human-wt/ZMB/UFS-NGS-MRC-DPRU1729/2012/G2P4	100.0																												
3	RVA/Human-wt/ZMB/UFS-NGS-MRC-DPRU4691/2014/G2P4	99.9	99.9																											
4	RVA/Human-wt/ZMB/UFS-NGS-MRC-DPRU4699/2014/G2P4	100.0	100.0	99.9																										
5	RVA/Human-wt/ZMB/UFS-NGS-MRC-DPRU4705/2014/G2P4	99.9	99.9	100.0	99.9																									
6	RVA/Human-wt/ZMB/UFS-NGS-MRC-DPRU4714/2014/G2P4	99.9	99.9	100.0	99.9	100.0																								
7	RVA/Human-wt/ZMB/UFS-NGS-MRC-DPRU4736/2014/G2P4	100.0	100.0	99.9	100.0	99.9	99.9																							
8	RVA/Human-wt/ZMB/UFS-NGS-MRC-DPRU4736_b/2014/G2P4	100.0	100.0	99.9	100.0	99.9	99.9	100.0																						
9	RVA/Human-wt/ZMB/UFS-NGS-MRC-DPRU4770/2014/G2P4	100.0	100.0	99.9	100.0	99.9	99.9	100.0	100.0																					
10	RVA/Human-wt/ZMB/UFS-NGS-MRC-DPRU4819/2014/G2P4	99.8	99.8	99.9	99.8	99.9	99.9	99.8	99.8	99.8																				
11	RVA/Human-wt/ZMB/UFS-NGS-MRC-DPRU9393/2015/G2P4	99.9	99.9	99.8	99.9	99.8	99.8	99.9	99.9	99.9	99.7																			
12	RVA/Human-wt/ZMB/UFS-NGS-MRC-DPRU9420/2015/G2P4	99.8	99.8	99.7	99.8	99.7	99.7	99.8	99.8	99.8	99.6	99.9																		
13	RVA/Human-wt/ZMB/UFS-NGS-MRC-DPRU9438/2015/G2P4	99.8	99.8	99.7	99.8	99.7	99.7	99.8	99.8	99.8	99.6	99.9	99.8																	
14	RVA/Human-wt/ZMB/UFS-NGS-MRC-DPRU9464/2015/G2P4	99.9	99.9	99.8	99.9	99.8	99.8	99.9	99.9	99.9	99.7	100.0	99.9	99.9																
15	RVA/Human-wt/ZMB/UFS-NGS-MRC-DPRU9475/2015/G2P4	99.9	99.9	99.8	99.9	99.8	99.8	99.9	99.9	99.9	99.7	100.0	99.9	99.9	100.0															
16	RVA/Human-wt/ZMB/UFS-NGS-MRC-DPRU9475b/2015/G2P4	99.9	99.9	99.8	99.9	99.8	99.8	99.9	99.9	99.9	99.7	100.0	99.9	99.9	100.0	100.0														
17	RVA/Human-wt/ZMB/UFS-NGS-MRC-DPRU9502/2015/G2P4	99.9	99.9	99.8	99.9	99.8	99.8	99.9	99.9	99.9	99.7	100.0	99.9	99.9	100.0	100.0	100.0													
18	RVA/Human-wt/ZMB/UFS-NGS-MRC-DPRU9503/2015/G2P4	99.9	99.9	99.8	99.9	99.8	99.8	99.9	99.9	99.9	99.7	100.0	99.9	99.9	100.0	100.0	100.0	100.0												
19	RVA/Human-wt/ZMB/UFS-NGS-MRC-DPRU13301/2016/G2P4	99.5	99.5	99.4	99.5	99.4	99.4	99.5	99.5	99.5	99.2	99.6	99.5	99.5	99.6	99.6	99.6	99.6	99.6											
20	RVA/Human-wt/ZMB/UFS-NGS-MRC-DPRU9515/2015/G2P4	99.9	99.9	99.8	99.9	99.8	99.8	99.9	99.9	99.9	99.7	100.0	99.9	99.9	100.0	100.0	100.0	100.0	100.0	99.6										
21	RVA/Human-wt/ZMB/UFS-NGS-MRC-DPRU9556/2015/G2P4	99.8	99.8	99.7	99.8	99.7	99.7	99.8	99.8	99.8	99.6	99.9	99.8	99.8	99.9	99.9	99.9	99.9	99.9	99.5	99.9									
22	RVA/Human-wt/ZMB/UFS-NGS-MRC-DPRU9559/2015/G2P4	99.8	99.8	99.7	99.8	99.7	99.7	99.8	99.8	99.8	99.6	99.9	99.8	99.8	99.9	99.9	99.9	99.9	99.9	99.5	99.9	99.8								
23	RVA/Human-wt/ZMB/UFS-NGS-MRC-DPRU13231/2016/G2P4	99.8	99.8	99.7	99.8	99.7	99.7	99.8	99.8	99.8	99.6	99.9	99.8	99.8	99.9	99.9	99.9	99.9	99.9	99.7	99.9	99.8	99.8							
24	RVA/Human-wt/ZMB/UFS-NGS-MRC-DPRU13352/2016/G2P4	99.9	99.9	99.8	99.9	99.8	99.8	99.9	99.9	99.9	99.7	100.0	99.9	99.9	100.0	100.0	100.0	100.0	100.0	99.6	100.0	99.9	99.9	99.9						
25	RVA/Human-wt/ZMB/UFS-NGS-MRC-DPRU13545/2016/G2P4	99.7	99.7	99.6	99.7	99.6	99.6	99.7	99.7	99.7	99.5	99.8	99.7	99.7	99.8	99.8	99.8	99.8	99.8	99.8	99.8	99.7	99.7	99.9	99.8					
26	RVA/Human-wt/ZMB/UFS-NGS-MRC-DPRU166599/2016/G2P4	99.8	99.8	99.7	99.8	99.7	99.7	99.8	99.8	99.8	99.6	99.9	99.8	99.8	99.9	99.9	99.9	99.9	99.9	99.5	99.9	99.8	99.8	99.8	99.9	99.7				
27	RVA/Human-wt/ZMB/UFS-NGS-MRC-DPRU16601/2016/G2P4	99.8	99.8	99.7	99.8	99.7	99.7	99.8	99.8	99.8	99.6	99.9	99.8	99.8	99.9	99.9	99.9	99.9	99.9	99.5	99.9	99.8	99.8	99.8	99.9	99.7	100.0			
28	RVA/Human-wt/ZMB/UFS-NGS-MRC-DPRU16639/2016/G2P4	99.8	99.8	99.7	99.8	99.7	99.7	99.8	99.8	99.8	99.6	99.9	99.8	99.8	99.9	99.9	99.9	99.9	99.9	99.5	99.9	99.8	99.8	99.8	99.9	99.7	100.0	100.0		
29	RVA/Human-wt/ZMB/UFS-NGS-MRC-DPRU16646/2016/G2P4	99.9	99.9	99.8	99.9	99.8	99.8	99.9	99.9	99.9	99.7	100.0	99.9	99.9	100.0	100.0	100.0	100.0	100.0	99.6	100.0	99.9	99.9	99.9	100.0	99.8	99.9	99.9	99.9	

NSP4

		1	2	3	4	5	6	7	8	9	10	11	12	13	14	15	16	17	18	19	20	21	22	23	24	25	26	27	28	29
1	RVA/Human-wt/ZMB/UFS-NGS-MRC-DPRU1724/2012/G2P4																													
2	RVA/Human-wt/ZMB/UFS-NGS-MRC-DPRU1729/2012/G2P4	100.0																												
3	RVA/Human-wt/ZMB/UFS-NGS-MRC-DPRU4691/2014/G2P4	99.8	99.8																											
4	RVA/Human-wt/ZMB/UFS-NGS-MRC-DPRU4699/2014/G2P4	100.0	100.0	99.8																										
5	RVA/Human-wt/ZMB/UFS-NGS-MRC-DPRU4705/2014/G2P4	99.6	99.6	99.8	99.6																									
6	RVA/Human-wt/ZMB/UFS-NGS-MRC-DPRU4714/2014/G2P4	99.8	99.8	100.0	99.8	99.8																								
7	RVA/Human-wt/ZMB/UFS-NGS-MRC-DPRU4718/2014/G2P4	99.8	99.8	100.0	99.8	99.8	100.0																							
8	RVA/Human-wt/ZMB/UFS-NGS-MRC-DPRU4736/2014/G2P4	100.0	100.0	99.8	100.0	99.6	99.8	99.8																						
9	RVA/Human-wt/ZMB/UFS-NGS-MRC-DPRU4770/2014/G2P4	100.0	100.0	99.8	100.0	99.6	99.8	99.8	100.0																					
10	RVA/Human-wt/ZMB/UFS-NGS-MRC-DPRU4819/2014/G2P4	99.6	99.6	99.8	99.6	99.6	99.8	99.8	99.6	99.6																				
11	RVA/Human-wt/ZMB/UFS-NGS-MRC-DPRU9393/2015/G2P4	99.4	99.4	99.2	99.4	99.0	99.2	99.2	99.4	99.4	99.0																			
12	RVA/Human-wt/ZMB/UFS-NGS-MRC-DPRU9475/2015/G2P4	99.2	99.2	99.0	99.2	98.9	99.0	99.0	99.2	99.2	98.9	99.8																		
13	RVA/Human-wt/ZMB/UFS-NGS-MRC-DPRU9495/2015/G2P4	99.4	99.4	99.2	99.4	99.0	99.2	99.2	99.4	99.4	99.0	100.0	99.8																	
14	RVA/Human-wt/ZMB/UFS-NGS-MRC-DPRU9420/2015/G2P4	99.4	99.4	99.2	99.4	99.0	99.2	99.2	99.4	99.4	99.0	100.0	99.8	100.0																
15	RVA/Human-wt/ZMB/UFS-NGS-MRC-DPRU9438/2015/G2P4	99.2	99.2	99.0	99.2	98.9	99.0	99.0	99.2	99.2	98.9	99.8	100.0	99.8	99.8															
16	RVA/Human-wt/ZMB/UFS-NGS-MRC-DPRU9464/2015/G2P4	99.4	99.4	99.2	99.4	99.0	99.2	99.2	99.4	99.4	99.0	100.0	99.8	100.0	100.0	99.8														
17	RVA/Human-wt/ZMB/UFS-NGS-MRC-DPRU9502/2015/G2P4	99.4	99.4	99.2	99.4	99.0	99.2	99.2	99.4	99.4	99.0	100.0	99.8	100.0	100.0	99.8	100.0													
18	RVA/Human-wt/ZMB/UFS-NGS-MRC-DPRU9503/2015/G2P4	99.4	99.4	99.2	99.4	99.0	99.2	99.2	99.4	99.4	99.0	100.0	99.8	100.0	100.0	99.8	100.0	100.0												
19	RVA/Human-wt/ZMB/UFS-NGS-MRC-DPRU9515/2015/G2P4	99.4	99.4	99.2	99.4	99.0	99.2	99.2	99.4	99.4	99.0	100.0	99.8	100.0	100.0	99.8	100.0	100.0	100.0											
20	RVA/Human-wt/ZMB/UFS-NGS-MRC-DPRU9556/2015/G2P4	99.2	99.2	99.0	99.2	98.9	99.0	99.0	99.2	99.2	98.9	99.8	99.6	99.8	99.8	99.6	99.8	99.8	99.8	99.8										
21	RVA/Human-wt/ZMB/UFS-NGS-MRC-DPRU9559/2015/G2P4	99.0	99.0	98.9	99.0	98.7	98.9	98.9	99.0	99.0	98.7	99.6	99.4	99.6	99.6	99.4	99.6	99.6	99.6	99.6	99.4									
22	RVA/Human-wt/ZMB/UFS-NGS-MRC-DPRU13231/2016/G2P4	99.4	99.4	99.2	99.4	99.0	99.2	99.2	99.4	99.4	99.0	100.0	99.8	100.0	100.0	99.8	100.0	100.0	100.0	100.0	99.8	99.6								
23	RVA/Human-wt/ZMB/UFS-NGS-MRC-DPRU13301/2016/G2P4	99.2	99.2	99.0	99.2	98.9	99.0	99.0	99.2	99.2	98.9	99.8	99.6	99.8	99.8	99.6	99.8	99.8	99.8	99.8	99.6	99.4	99.8							
24	RVA/Human-wt/ZMB/UFS-NGS-MRC-DPRU13352/2016/G2P4	99.2	99.2	99.0	99.2	98.9	99.0	99.0	99.2	99.2	98.9	99.8	99.6	99.8	99.8	99.6	99.8	99.8	99.8	99.8	99.6	99.4	99.8	99.6						
25	RVA/Human-wt/ZMB/UFS-NGS-MRC-DPRU13545/2016/G2P4	99.2	99.2	99.0	99.2	98.9	99.0	99.0	99.2	99.2	98.9	99.8	99.6	99.8	99.8	99.6	99.8	99.8	99.8	99.8	99.6	99.4	99.8	99.6	99.6					
26	RVA/Human-wt/ZMB/UFS-NGS-MRC-DPRU16599/2016/G2P4	99.2	99.2	99.0	99.2	98.9	99.0	99.0	99.2	99.2	98.9	99.8	99.6	99.8	99.8	99.6	99.8	99.8	99.8	99.8	99.6	99.4	99.8	99.6	99.6	99.6				
27	RVA/Human-wt/ZMB/UFS-NGS-MRC-DPRU16601/2016/G2P4	99.2	99.2	99.0	99.2	98.9	99.0	99.0	99.2	99.2	98.9	99.8	99.6	99.8	99.8	99.6	99.8	99.8	99.8	99.8	99.6	99.4	99.8	99.6	99.6	99.6	100.0			
28	RVA/Human-wt/ZMB/UFS-NGS-MRC-DPRU16639/2016/G2P4	99.2	99.2	99.0	99.2	98.9	99.0	99.0	99.2	99.2	98.9	99.8	99.6	99.8	99.8	99.6	99.8	99.8	99.8	99.8	99.6	99.4	99.8	99.6	99.6	99.6	100.0	100.0		
29	RVA/Human-wt/ZMB/UFS-NGS-MRC-DPRU16646/2016/G2P4	99.2	99.2	99.0	99.2	98.9	99.0	99.0	99.2	99.2	98.9	99.8	99.6	99.8	99.8	99.6	99.8	99.8	99.8	99.8	99.6	99.4	99.8	99.6	100.0	99.6	99.6	99.6	99.6	

NSP5

		1	2	3	4	5	6	7	8	9	10	11	12	13	14	15	16	17	18	19	20	21	22	23	24	25	26	27	28	29
**1**	RVA/Human-wt/ZMB/UFS-NGS-MRC-DPRU1724/2012/G2P4																													
**2**	RVA/Human-wt/ZMB/UFS-NGS-MRC-DPRU1729/2012/G2P4	99.8																												
**3**	RVA/Human-wt/ZMB/UFS-NGS-MRC-DPRU4819/2014/G2P4	100.0	99.8																											
**4**	RVA/Human-wt/ZMB/UFS-NGS-MRC-DPRU4691/2014/G2P4	100.0	99.8	100.0																										
**5**	RVA/Human-wt/ZMB/UFS-NGS-MRC-DPRU4699/2014/G2P4	99.8	99.7	99.8	99.8																									
**6**	RVA/Human-wt/ZMB/UFS-NGS-MRC-DPRU4705/2014/G2P4	99.8	99.7	99.8	99.8	99.7																								
**7**	RVA/Human-wt/ZMB/UFS-NGS-MRC-DPRU4714/2014/G2P4	99.8	99.7	99.8	99.8	99.7	99.7																							
**8**	RVA/Human-wt/ZMB/UFS-NGS-MRC-DPRU4718/2014/G2P4	99.8	99.7	99.8	99.8	99.7	99.7	99.7																						
**9**	RVA/Human-wt/ZMB/UFS-NGS-MRC-DPRU4736/2014/G2P4	99.8	99.7	99.8	99.8	100.0	99.7	99.7	99.7																					
**10**	RVA/Human-wt/ZMB/UFS-NGS-MRC-DPRU4770/2014/G2P4	99.8	99.7	99.8	99.8	100.0	99.7	99.7	99.7	100.0																				
**11**	RVA/Human-wt/ZMB/UFS-NGS-MRC-DPRU9420/2015/G2P4	99.7	99.5	99.7	99.7	99.5	99.8	99.5	99.5	99.5	99.5																			
**12**	RVA/Human-wt/ZMB/UFS-NGS-MRC-DPRU9438/2015/G2P4	99.5	99.3	99.5	99.5	99.3	99.7	99.3	99.5	99.3	99.3	99.8																		
**13**	RVA/Human-wt/ZMB/UFS-NGS-MRC-DPRU9464/2015/G2P4	99.7	99.5	99.7	99.7	99.5	99.8	99.5	99.5	99.5	99.5	100.0	99.8																	
**14**	RVA/Human-wt/ZMB/UFS-NGS-MRC-DPRU9475/2015/G2P4	99.5	99.3	99.5	99.5	99.3	99.7	99.3	99.5	99.3	99.3	99.8	100.0	99.8																
**15**	RVA/Human-wt/ZMB/UFS-NGS-MRC-DPRU9495/2015/G2P4	99.7	99.5	99.7	99.7	99.5	99.8	99.5	99.5	99.5	99.5	100.0	99.8	100.0	99.8															
**16**	RVA/Human-wt/ZMB/UFS-NGS-MRC-DPRU9502/2015/G2P4	99.7	99.5	99.7	99.7	99.5	99.8	99.5	99.5	99.5	99.5	100.0	99.8	100.0	99.8	100.0														
**17**	RVA/Human-wt/ZMB/UFS-NGS-MRC-DPRU9503/2015/G2P4	99.7	99.5	99.7	99.7	99.5	99.8	99.5	99.5	99.5	99.5	100.0	99.8	100.0	99.8	100.0	100.0													
**18**	RVA/Human-wt/ZMB/UFS-NGS-MRC-DPRU9515/2015/G2P4	99.7	99.5	99.7	99.7	99.5	99.8	99.5	99.5	99.5	99.5	100.0	99.8	100.0	99.8	100.0	100.0	100.0												
**19**	RVA/Human-wt/ZMB/UFS-NGS-MRC-DPRU9556/2015/G2P4	99.5	99.3	99.5	99.5	99.3	99.7	99.3	99.3	99.3	99.3	99.8	99.7	99.8	99.7	99.8	99.8	99.8	99.8											
**20**	RVA/Human-wt/ZMB/UFS-NGS-MRC-DPRU9559/2015/G2P4	99.7	99.5	99.7	99.7	99.5	99.8	99.5	99.5	99.5	99.5	100.0	99.8	100.0	99.8	100.0	100.0	100.0	100.0	99.8										
**21**	RVA/Human-wt/ZMB/UFS-NGS-MRC-DPRU13231/2016/G2P4	99.7	99.5	99.7	99.7	99.5	99.8	99.5	99.5	99.5	99.5	100.0	99.8	100.0	99.8	100.0	100.0	100.0	100.0	99.8	100.0									
**22**	RVA/Human-wt/ZMB/UFS-NGS-MRC-DPRU13352/2016/G2P4	99.7	99.5	99.7	99.7	99.5	99.8	99.5	99.5	99.5	99.5	100.0	99.8	100.0	99.8	100.0	100.0	100.0	100.0	99.8	100.0	100.0								
**23**	RVA/Human-wt/ZMB/UFS-NGS-MRC-DPRU13545/2016/G2P4	99.7	99.5	99.7	99.7	99.5	99.8	99.5	99.5	99.5	99.5	100.0	99.8	100.0	99.8	100.0	100.0	100.0	100.0	99.8	100.0	100.0	100.0							
**24**	RVA/Human-wt/ZMB/UFS-NGS-MRC-DPRU16599/2016/G2P4	99.7	99.5	99.7	99.7	99.5	99.8	99.5	99.5	99.5	99.5	100.0	99.8	100.0	99.8	100.0	100.0	100.0	100.0	99.8	100.0	100.0	100.0	100.0						
**25**	RVA/Human-wt/ZMB/UFS-NGS-MRC-DPRU16601/2016/G2P4	99.7	99.5	99.7	99.7	99.5	99.8	99.5	99.5	99.5	99.5	100.0	99.8	100.0	99.8	100.0	100.0	100.0	100.0	99.8	100.0	100.0	100.0	100.0	100.0					
**26**	RVA/Human-wt/ZMB/UFS-NGS-MRC-DPRU16639/2016/G2P4	99.7	99.5	99.7	99.7	99.5	99.8	99.5	99.5	99.5	99.5	100.0	99.8	100.0	99.8	100.0	100.0	100.0	100.0	99.8	100.0	100.0	100.0	100.0	100.0	100.0				
**27**	RVA/Human-wt/ZMB/UFS-NGS-MRC-DPRU9393/2015/G2P4	99.7	99.5	99.7	99.7	99.5	99.8	99.5	99.5	99.5	99.5	100.0	99.8	100.0	99.8	100.0	100.0	100.0	100.0	99.8	100.0	100.0	100.0	100.0	100.0	100.0	100.0			
**28**	RVA/Human-wt/ZMB/UFS-NGS-MRC-DPRU13301/2016/G2P4	99.7	99.5	99.7	99.7	99.5	99.8	99.5	99.5	99.5	99.5	100.0	99.8	100.0	99.8	100.0	100.0	100.0	100.0	99.8	100.0	100.0	100.0	100.0	100.0	100.0	100.0	100.0		
**29**	RVA/Human-wt/ZMB/UFS-NGS-MRC-DPRU16646/2016/G2P4	99.5	99.3	99.5	99.5	99.3	99.7	99.7	99.3	99.3	99.3	99.8	99.7	99.8	99.7	99.8	99.8	99.8	99.8	99.7	99.8	99.8	99.8	99.8	99.8	99.8	99.8	99.8	99.8	
